# Supplementary material for: Chemoproteomic discovery of a human RNA ligase
Source: Nat Commun. 2023 Feb 15;14:842. doi: 10.1038/s41467-023-36451-x (PMC9931718; doi:10.1038/s41467-023-36451-x)
Supplement: Supplementary file 1 — Supplemental Information [file 41467_2023_36451_MOESM1_ESM.pdf]

Chemoproteomic discovery of a human RNA ligase

Authors

Yizhi Yuan[1,2], Florian M. Stumpf[1,2], Lisa A. Schlor[1,2], Olivia P. Schmidt[1], Philip Saumer[1,2], Luisa B. Huber[1,2], Matthias Frese[1,2], Eva Höllmüller[1,2], Martin Scheffner[2,3], Florian Stengel[2,3], Kay Diederichs[2,3], Andreas Marx[1,2]*

Affiliations

[1]Department of Chemistry, University of Konstanz; Universitätsstraße 10, 78457 Konstanz, Germany.

[2]Konstanz Research School Chemical Biology; University of Konstanz, Universitätsstraße 10, 78457 Konstanz, Germany.

[3]Department of Biology, University of Konstanz; Universitätsstraße 10, 78457 Konstanz, Germany.

*Corresponding author. Email: andreas.marx@uni-konstanz.de

Supplementary Information

Table of Contents

Supplementary Figures……………………………………………………………S3-S24
Supplementary Tables………………………………………………………………S25-S26
Supplementary Notes……………………………………………………………..S27-S28
Supplementary Methods……………………………………………………………S29-S55
Supplementary References…………………………………………………………….S56

Supplementary Figures

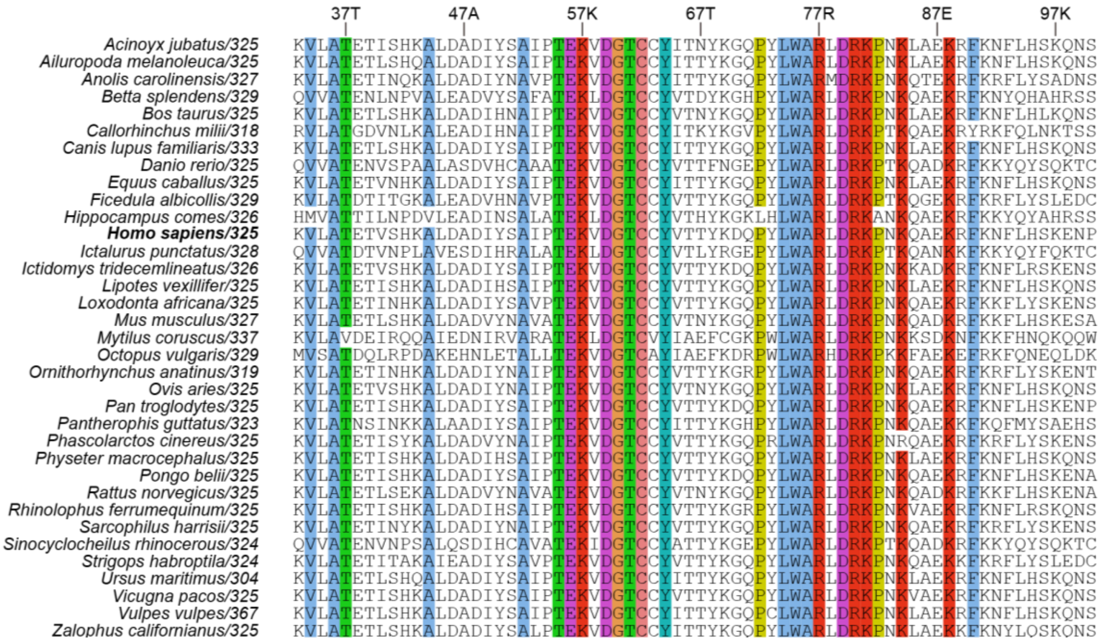

Supplementary Fig. 1

Multiple amino acid sequence alignment of C12orf29 homologues in different species. The amino acid sequence of C12orf29 of *Homo sapiens* (in bold) was used as reference. The number after the species names represents the length of C12orf29 homologues. Residues with >95% identities are shaded with different colors. Hydrophobic residues are in blue. Positively charged residues are in red. Negatively charged residues are in magenta. Polar residues are in green. Cysteines are in pink. Glycines are in orange. Prolines are in yellow. Aromatic residues are in cyan. The alignment was done with Clustal Omega[1] (retrieved on 2021, Jan 6th) and illustrated with Jalview[2] (version 2.11.1.1).

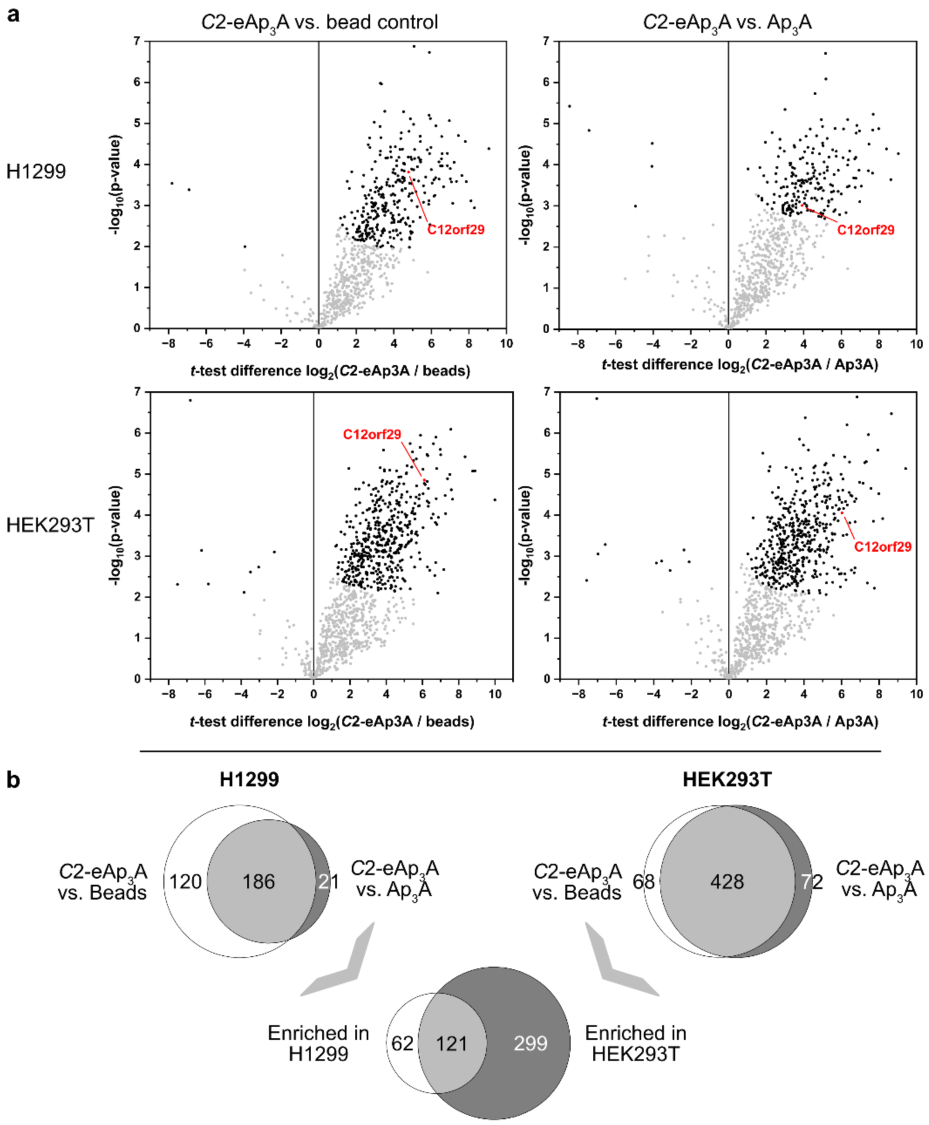

Supplementary Fig. 2

Results of chemical proteomics experiment. a, Volcano plots of pairwise comparisons between *C*2-eAp3A and bead control as well as *C*2-eAp3A and Ap3A for both cell types (H1299 and HEK293T). Black circles: significantly enriched proteins ($S_0 = 0.1$, FDR = 0.001), red circle: C12orf29. **b,** Venn diagrams of overlap between significantly enriched proteins of both Student's *t*-tests for each cell type (top) and Venn diagram of overlap between proteins that were significantly enriched in both Student's *t*-tests for each cell type and additionally showed a *t*-test difference > 1 (bottom). C12orf29 was significantly enriched for *C*2-eAp3A in all pairwise comparisons in both cell types. Venn diagrams were created using eulerr.co[3]. Chemical proteomics mass spectrometry data are provided as Supplementary Dataset and have been deposited to the ProteomeXchange Consortium via the PRIDE partner repository with the dataset identifier PXD038132.

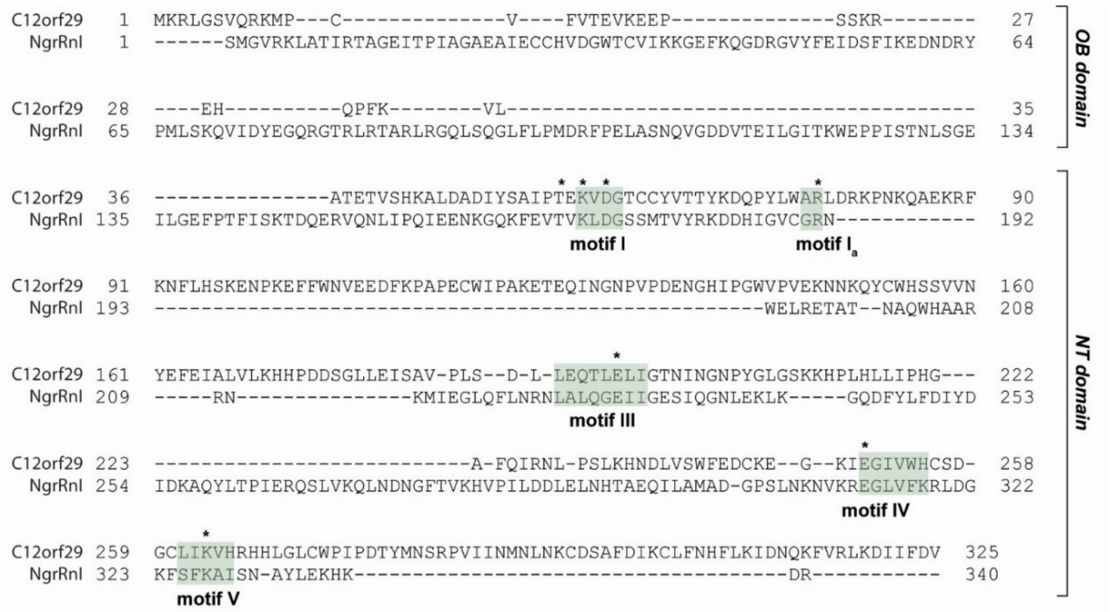

Supplementary Fig. 3

Structure-based amino acid sequence alignment of C12orf29 and *Ngr*Rnl. The OB domain and NT domain of *Ngr*Rnl are indicated by brackets at right. Motifs I, I_a, III, IV, and V are shaded green. Residues that are potentially essential for the ligase activity were indicated by asterisks. The alignment was done with PROMALS3D[4] using the C12orf29 model predicted by AlphaFold and *Ngr*Rnl with PDB ID: 5COT. OB, oligonucleotide-binding. NT, nucleotidyltransferase.

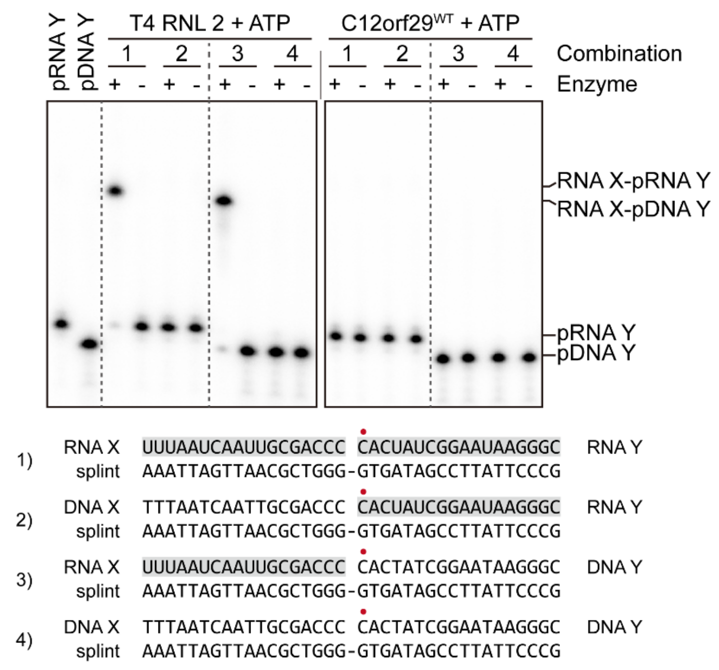

Supplementary Fig. 4

Non-reactive substrates for ligation. Four nicked duplexes with different combinations of DNA/RNA oligonucleotides were tested separately. RNA oligos were shaded in gray. The ^{32}P-labelled 5'-PO_4 were depicted in red. All reactions were performed under identical conditions: 1.0 µM C12orf29^{WT}, 0.1 µM 5' ^{32}P-labelled oligonucleotide, 200 µM ATP, 5.0 mM $MgCl_2$, and 1.0 mM DTT in 50 mM Tris-HOAc at pH 7.0 for 60 min at 37 ºC. T4 RNA ligase 2 was employed for the positive controls. The radioactive oligonucleotides were resolved by urea-PAGE and phosphorimaging (representative images of n = 3). Source data are provided as a Source Data file.

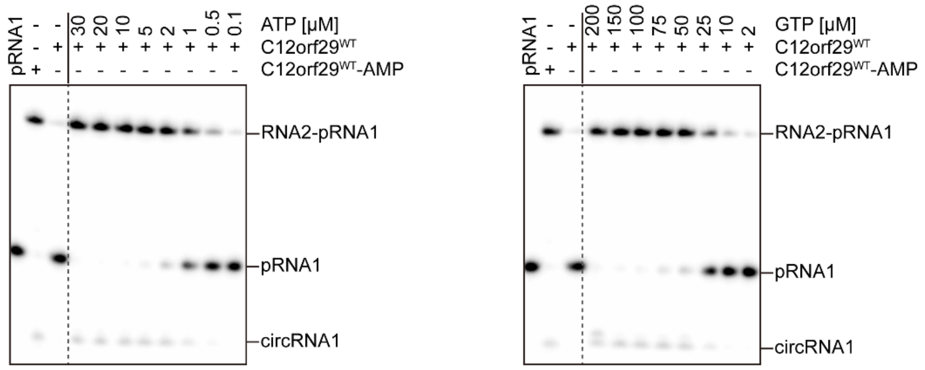

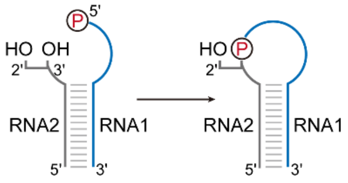

	ATP	GTP
V_{max} [nM/min]	1.68 ± 0.06	1.89 ± 0.11
K_M [µM]	1.30 ± 0.19	37.7 ± 7.26
k_{cat} [s^{-1}]	2.81×10^{-5} $\pm 9.88 \times 10^{-7}$	3.14×10^{-5} $\pm 1.90 \times 10^{-6}$
k_{cat}/K_M [s^{-1}/M]	21.6	0.83

Supplementary Fig. 5

Steady state kinetics using ATP or GTP as substrate. Non-cropped urea-PAGE analysis depicted in Fig. 3c were displayed in the top panel. Exemplary RNA ligation reactions depicted were performed with 1.0 µM C12orf29^{WT} for 60 min at 37 °C at varying concentrations of ATP or GTP. The rate of RNA2-pRNA1 formation was quantified by the intensities of the bands for each chamber. Values represented arithmetic mean ± SD from biological triplicates. Source data are provided as a Source Data file.

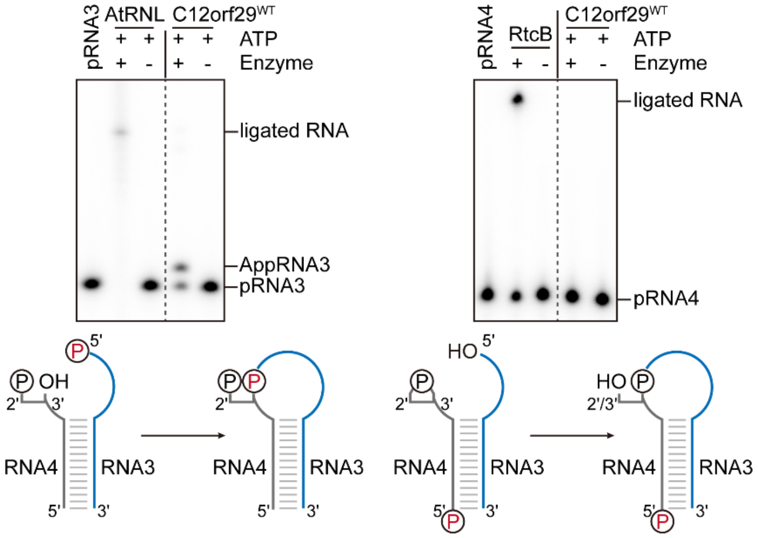

Supplementary Fig. 6

Substrate scope of C12orf29. Neither RNA termini modifications (2'-PO_4 or 2',3'-cPO_4) were not used as substrates for C12orf29. Radioactive 5'-PO_4 are shown in red. Representative images of n = 3 biological replicates.

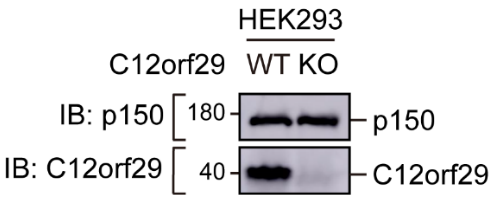

Supplementary Fig. 7

Immunoblotting analysis of C12orf29 expression in WT and *C12ORF29*-KO HEK293 cells. WT and *C12ORF29*-KO HEK293 cells were lysed in 1x loading buffer without β-mercaptoethanol, heated to 95 °C for 5 min and sonicated on ice. The lysates were centrifuged (12,000 x g, 5 min) and the protein concentrations of the supernatant were determined by BCA assay. 40 µg total protein were resolved by SDS-PAGE and subsequent immunoblotting as described above (representative images of n = 3). p150 was used as a loading control. Source data are provided as a Source Data file.

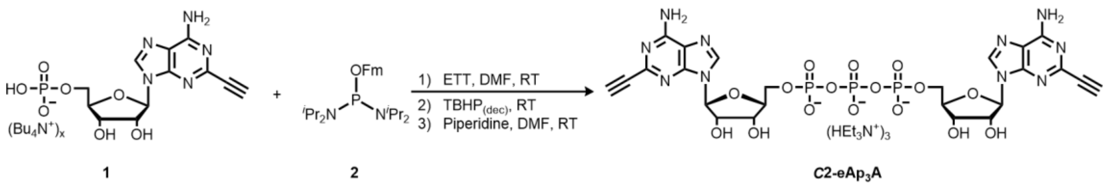

Supplementary Fig. 8

Synthesis of *C*2-eAp$_3$A.

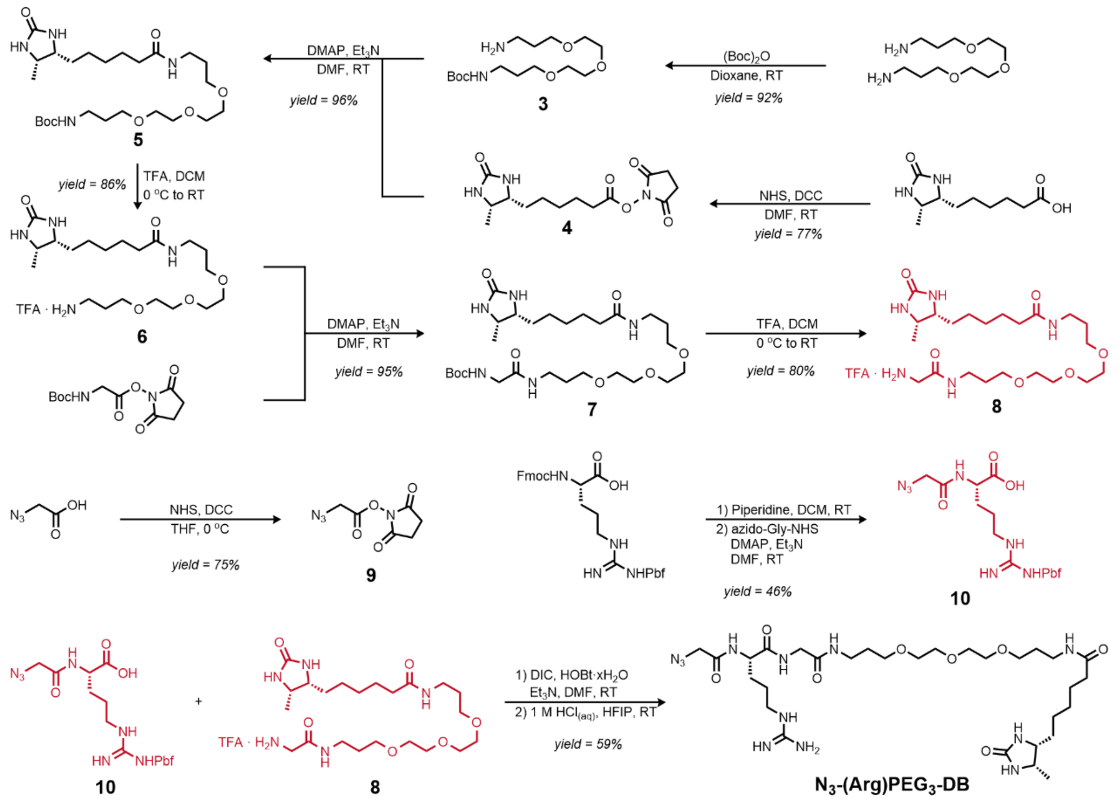

Supplementary Fig. 9

Overview of the synthesis of N3-(Arg)PEG3-DB. The synthesis was inspired by ref. 5.

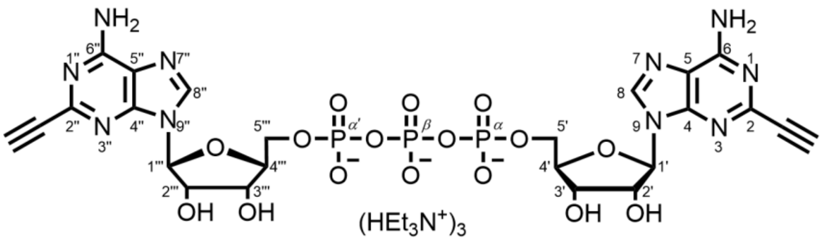

2,2''-Diethynyldiadenosine-*O*5',*O*5'''-triphosphate (triethylammonium salt form)

***C*2-eAp3A**

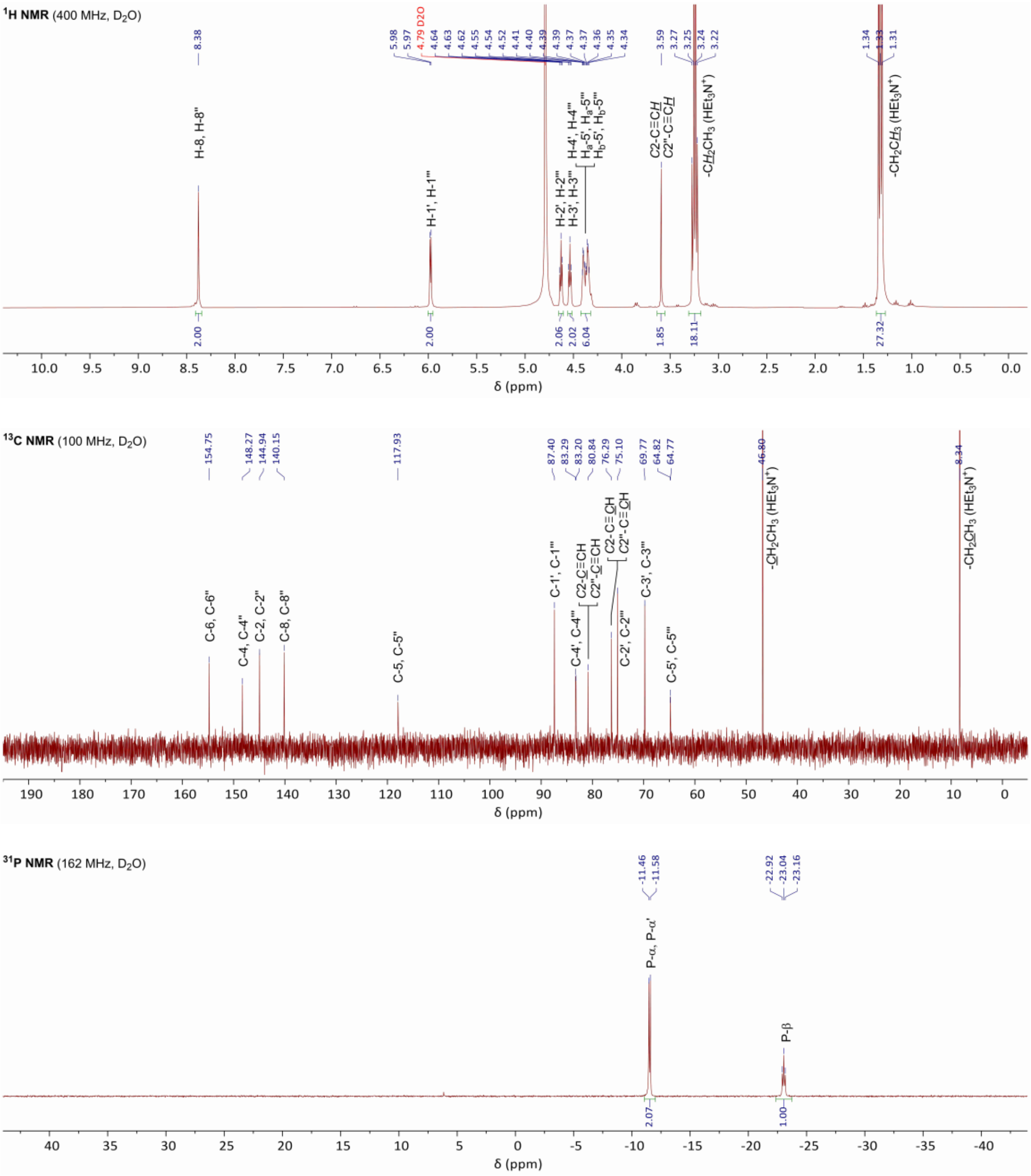

Supplementary Fig. 10

NMR characterisation of 2,2''-diethynyldiadenosine-*O*5',*O*5'''-triphosphate (triethyl-ammonium salt form) *C*2-eAp3A.

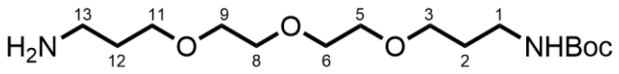

tert-Butyl (3-(2-(2-(3-aminopropoxy)ethoxy)ethoxy)propyl)carbamate **3**

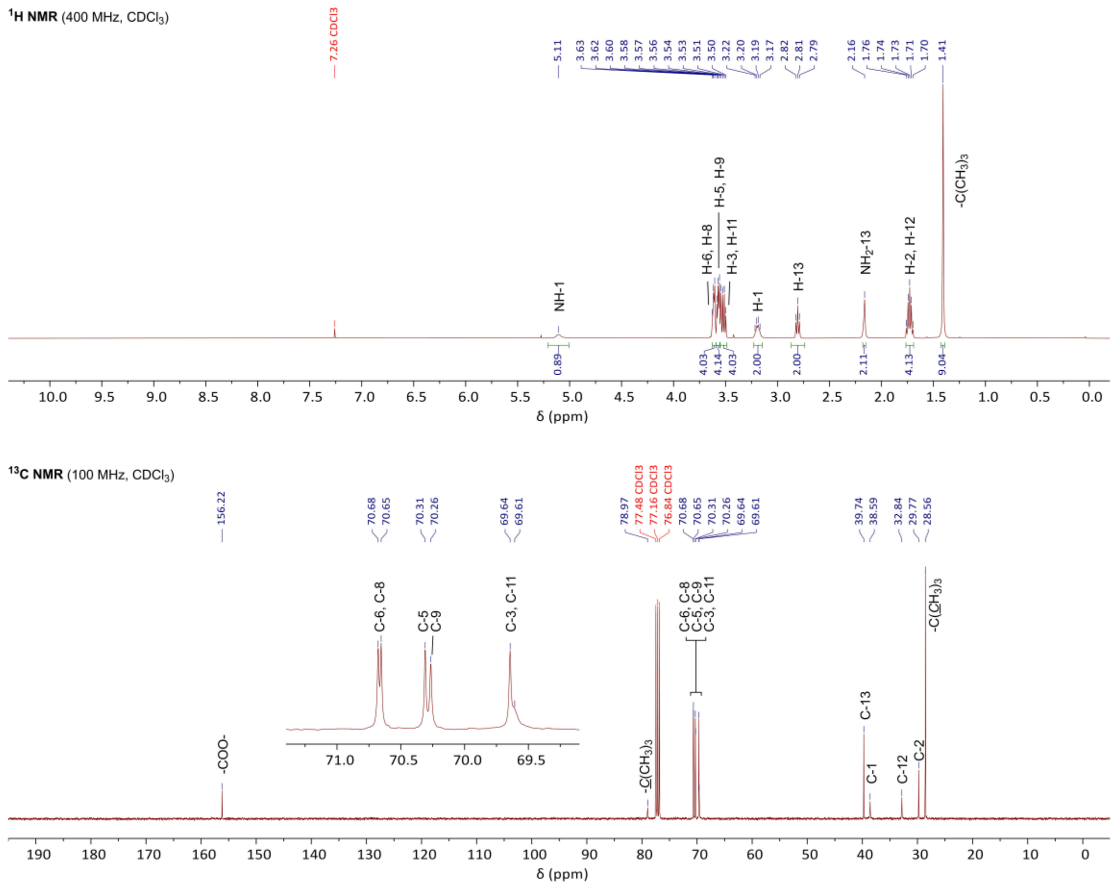

Supplementary Fig. 11

NMR characterisation of *tert*-butyl (3-(2-(2-(3-aminopropoxy)ethoxy)ethoxy)-propyl)carbamate (3).

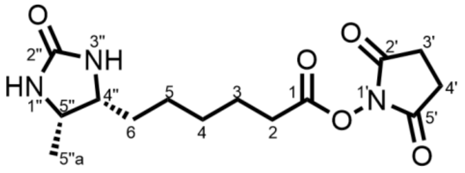

2,5-Dioxopyrrolidin-1-yl 6-((4*R*,5*S*)-5-methyl-2-oxoimidazolidin-4-yl)hexanoate **4**

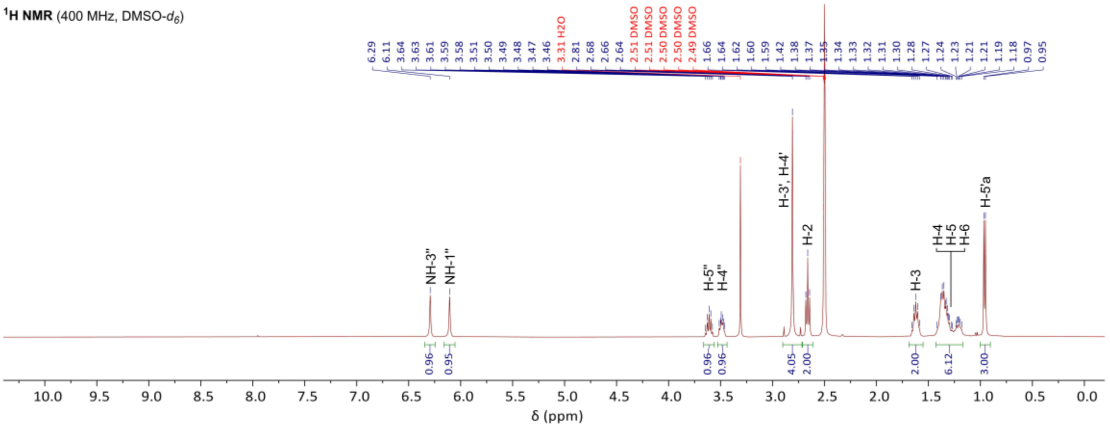

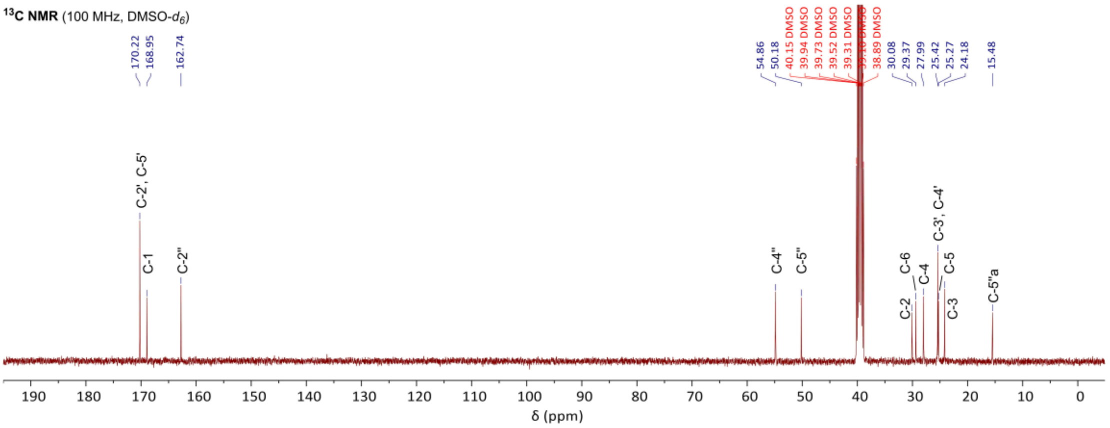

Supplementary Fig. 12

NMR characterisation of 2,5-dioxopyrrolidin-1-yl 6-((4*R*,5*S*)-5-methyl-2-oxoimidazolidin-4-yl)hexanoate (4).

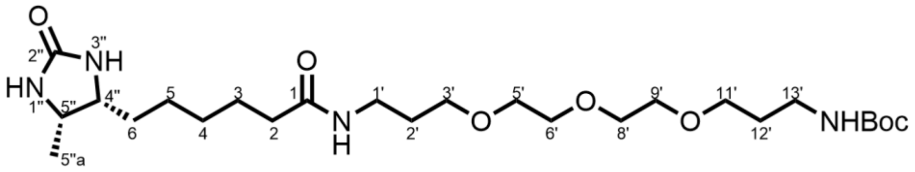

tert-Butyl (20-((4*R*,5*S*)-5-methyl-2-oxoimidazolidin-4-yl)-15-oxo-4,7,10-trioxa-14-azaicosyl)carbamate **5**

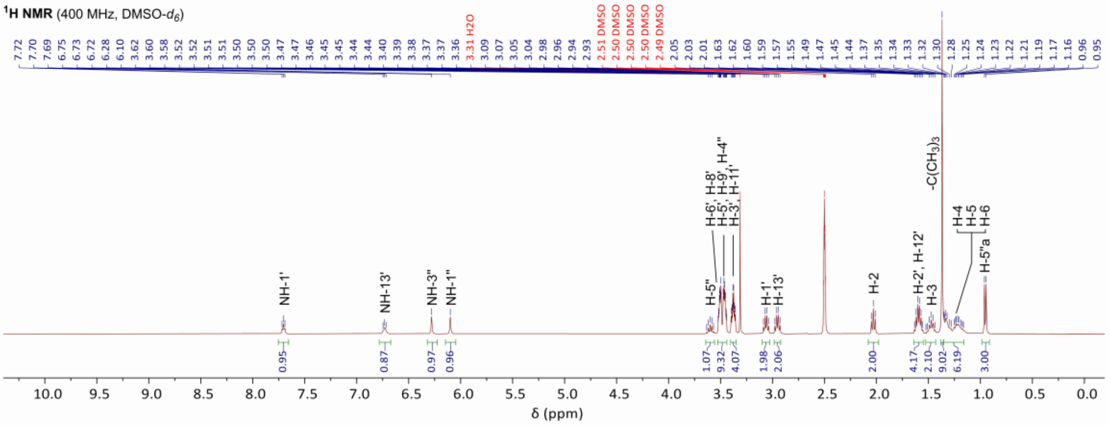

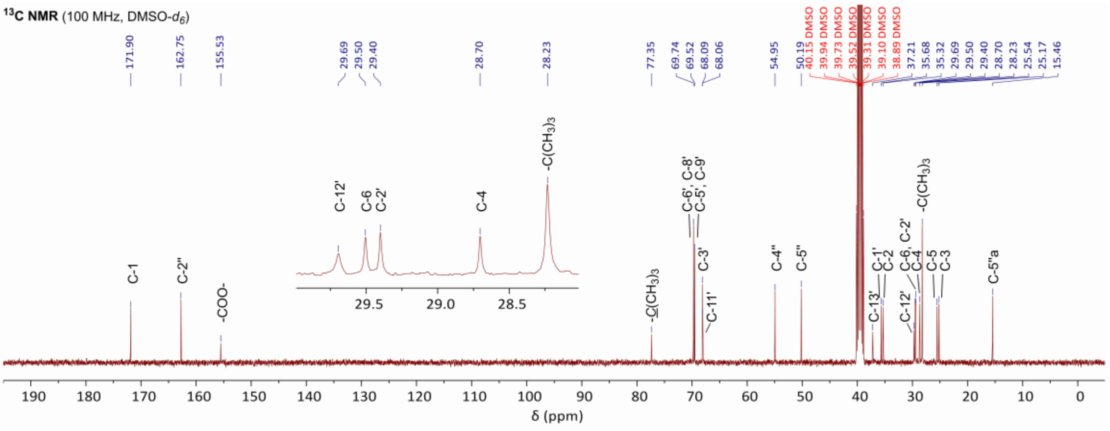

Supplementary Fig. 13

NMR characterisation of *tert*-butyl (20-((4*R*,5*S*)-5-methyl-2-oxoimidazolidin-4-yl)-15-oxo-4,7,10-trioxa-14-azaicosyl)carbamate (5).

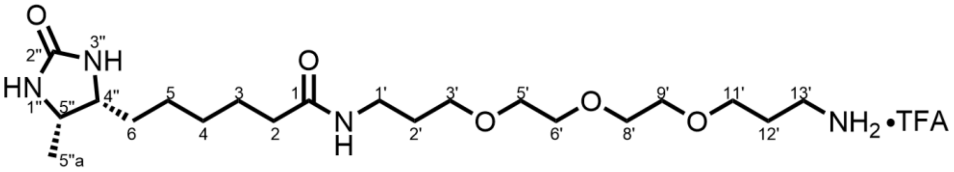

N-(3-(2-(2-(3-Aminopropoxy)ethoxy)ethoxy)propyl)-6-((4*R*,5*S*)-5-methyl-2-oxoimidazolidin-4-yl)hexanamide (trifluoroacetic acid salt form) **6**

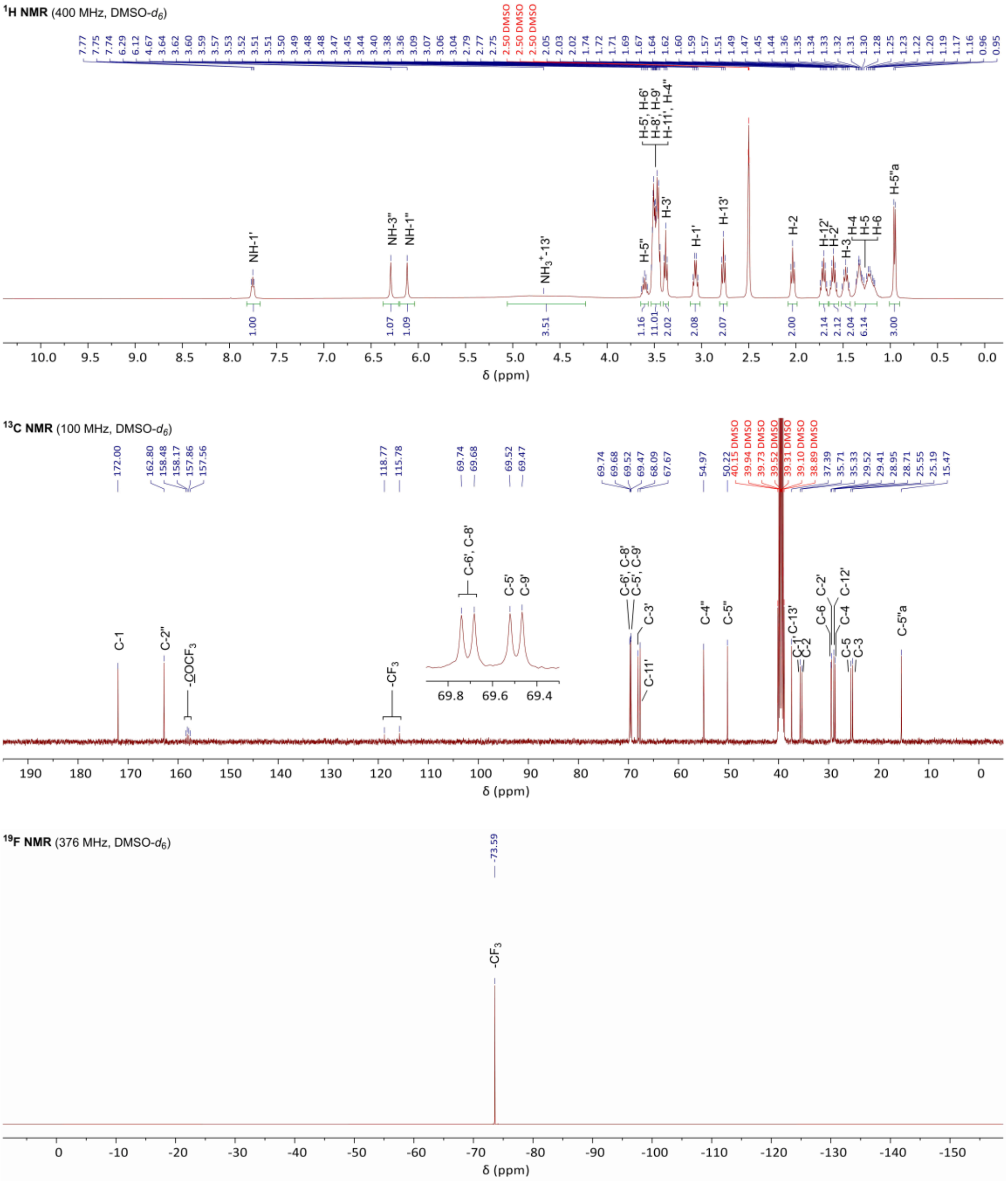

Supplementary Fig. 14

NMR characterisation of *N*-(3-(2-(2-(3-aminopropoxy)ethoxy)ethoxy)propyl)-6-((4*R*,5*S*)-5-methyl-2-oxoimidazolidin-4-yl)hexanamide (trifluoroacetic acid salt form) (6).

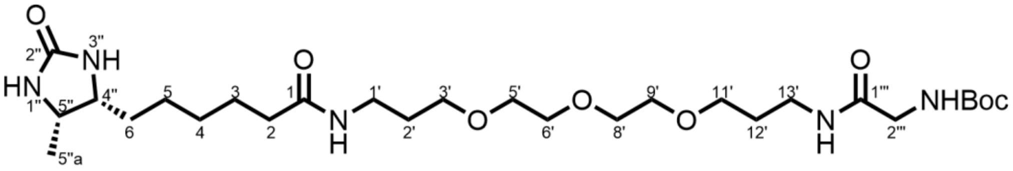

tert-Butyl (23-((4*R*,5*S*)-5-methyl-2-oxoimidazolidin-4-yl)-2,18-dioxo-7,10,13-trioxa-3,17-diazatricosyl)carbamate **7**

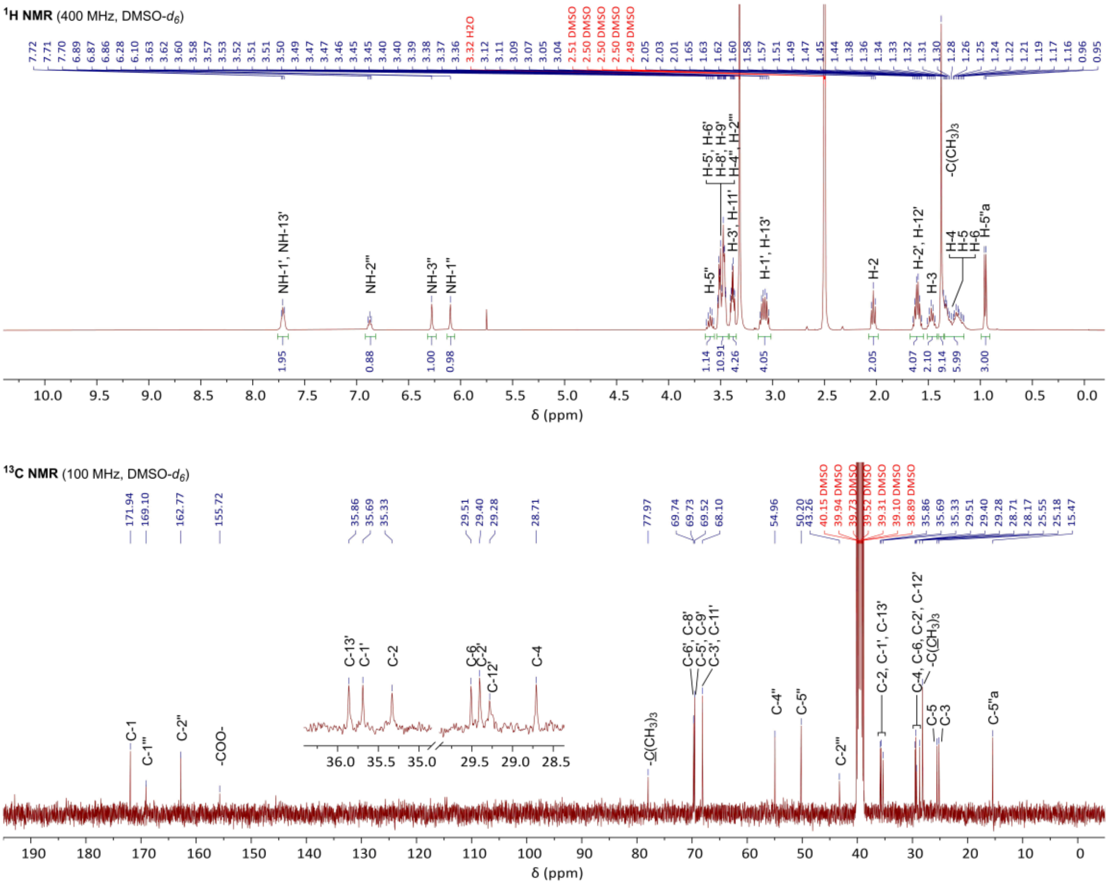

Supplementary Fig. 15

NMR characterisation of *tert*-butyl (23-((4*R*,5*S*)-5-methyl-2-oxoimidazolidin-4-yl)-2,18-dioxo-7,10,13-trioxa-3,17-diazatricosyl)carbamate (7).

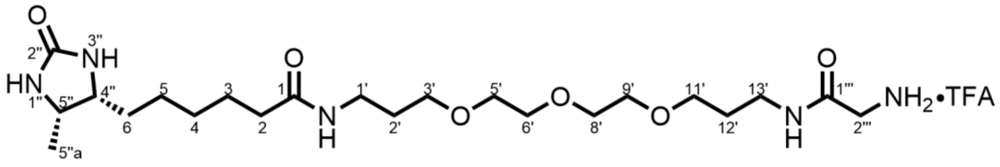

N-(1-Amino-2-oxo-7,10,13-trioxa-3-azahexadecan-16-yl)-6-((4*R*,5*S*)-5-methyl-2-oxoimidazolidin-4-yl)hexanamide (trifluoroacetic acid salt form) **8**

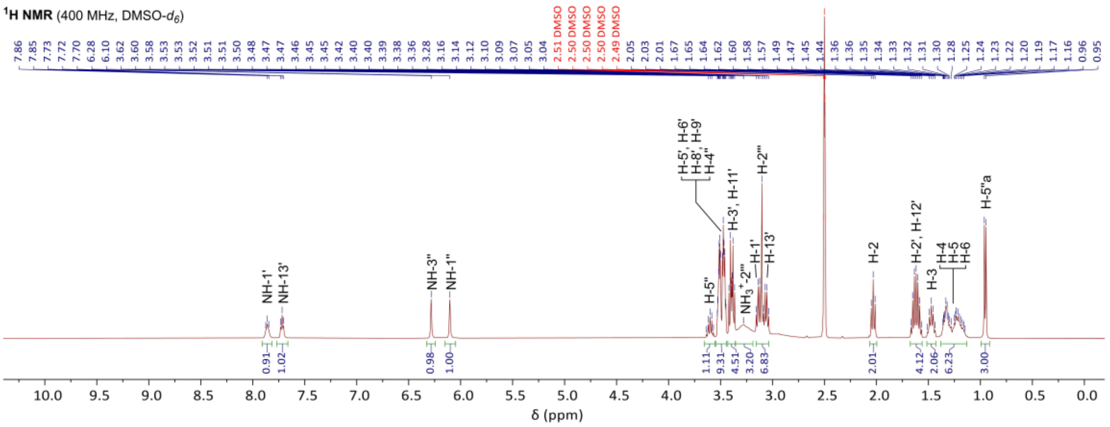

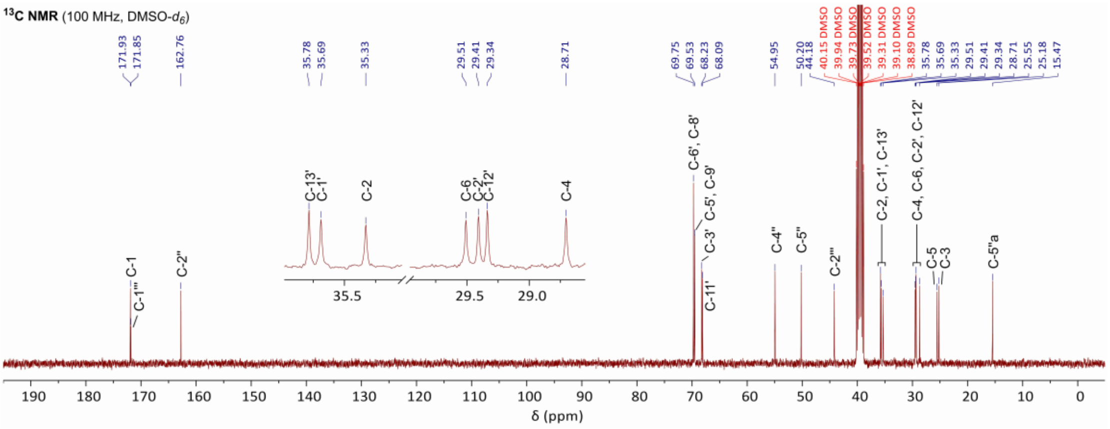

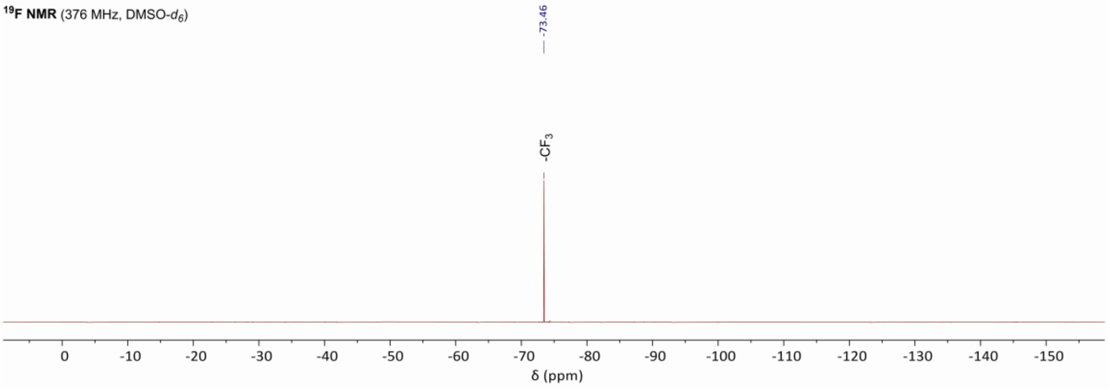

Supplementary Fig. 16

NMR characterisation of *N*-(1-amino-2-oxo-7,10,13-trioxa-3-azahexadecan-16-yl)-6-((4*R*,5*S*)-5-methyl-2-oxoimidazolidin-4-yl)hexanamide (trifluoroacetic acid salt form) (8).

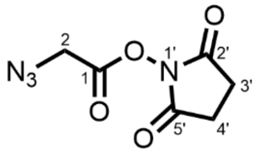

2,5-Dioxopyrrolidin-1-yl 2-azidoacetate **9**

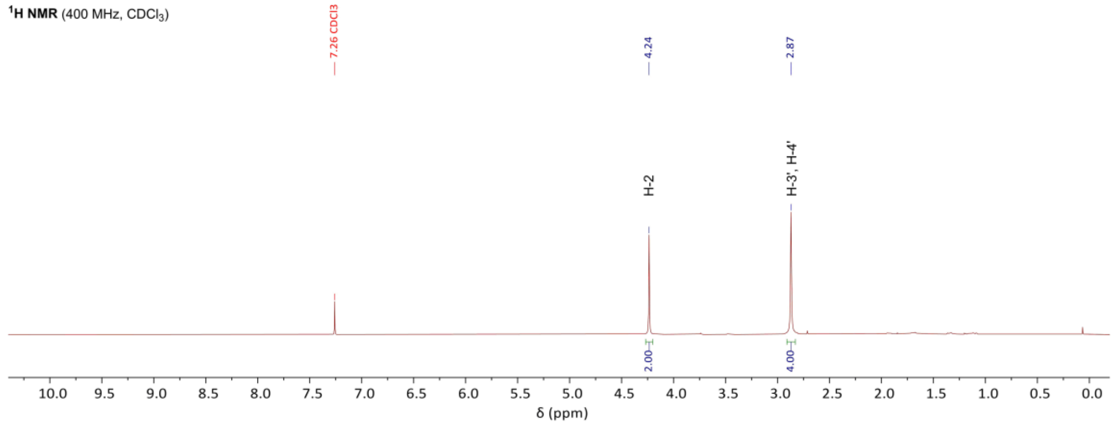

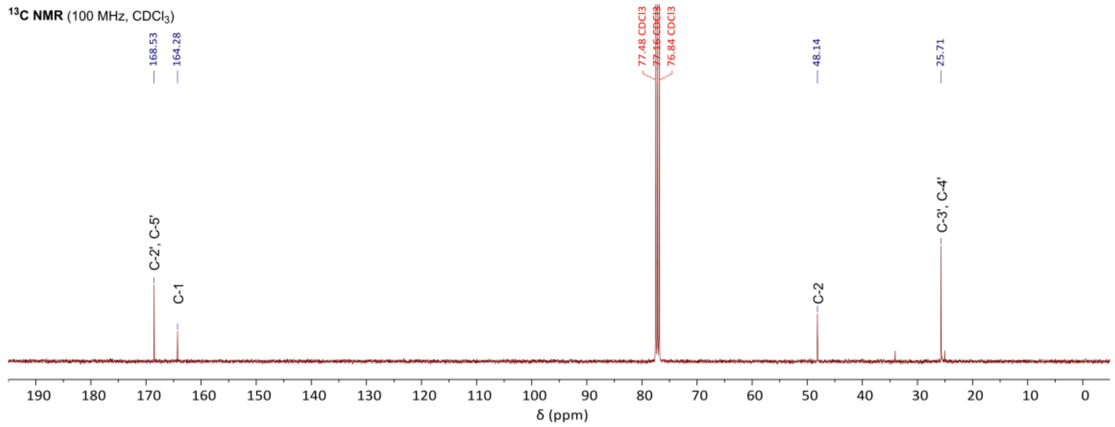

Supplementary Fig. 17

NMR characterisation of 2,5-dioxopyrrolidin-1-yl 2-azidoacetate (9).

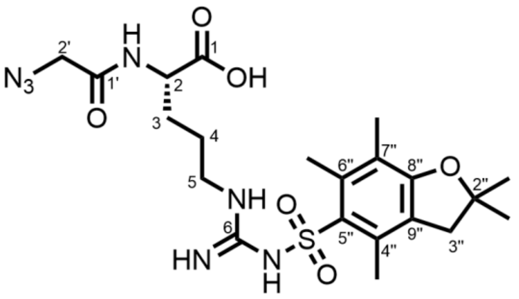

N^2-(2-Azidoacetyl)-N^{ω}-((2,2,4,6,7-pentamethyl-2,3-dihydrobenzofuran-5-yl)sulfonyl)-L-arginine **10**

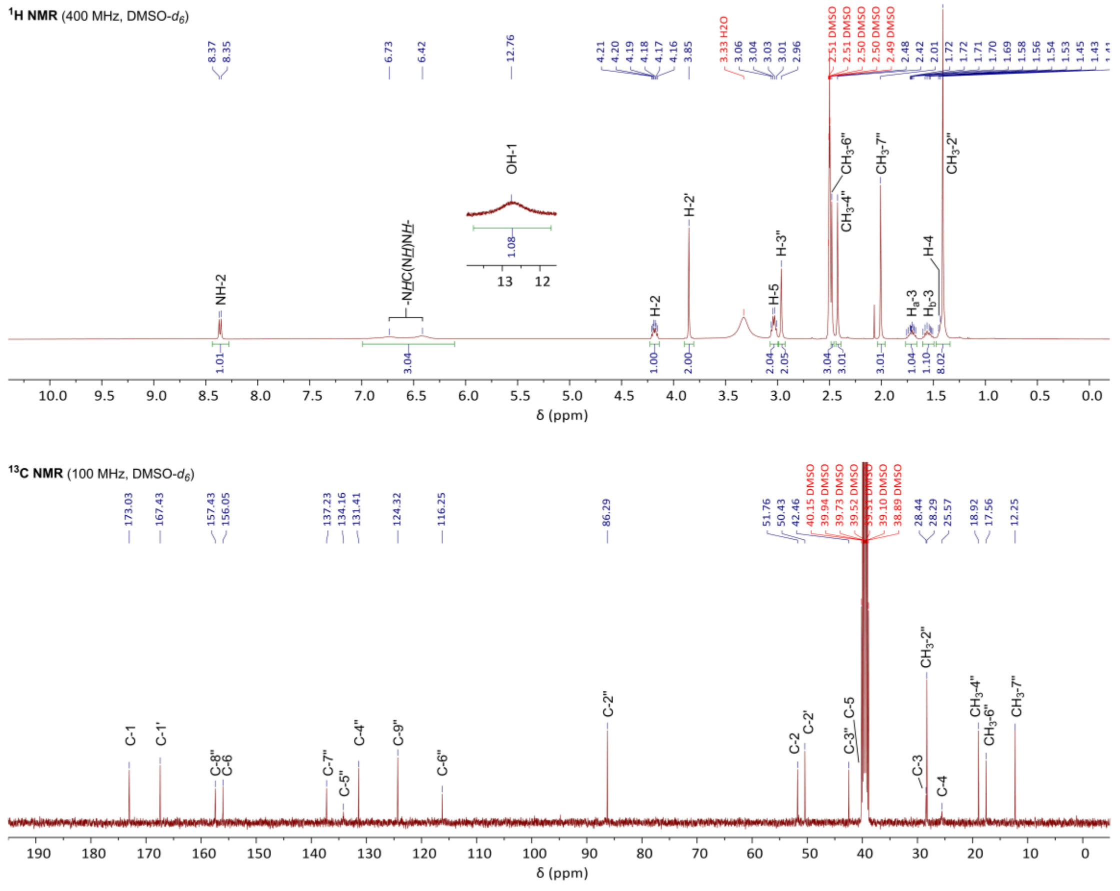

Supplementary Fig. 18

NMR characterisation of N^2-(2-azidoacetyl)-N^{ω}-((2,2,4,6,7-pentamethyl-2,3-dihydrobenzofuran-5-yl)sulfonyl)-L-arginine (10).

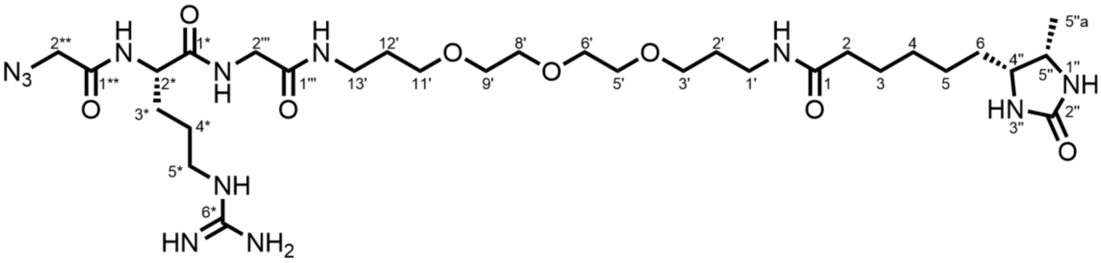

N-((*S*)-1-Amino-6-(2-azidoacetamido)-1-imino-7,10-dioxo-15,18,21-trioxa-2,8,11-triazatetracosan-24-yl)-6-((4*R*,5*S*)-5-methyl-2-oxoimidazolidin-4-yl)hexanamide

N_3-(Arg)PEG$_3$-DB

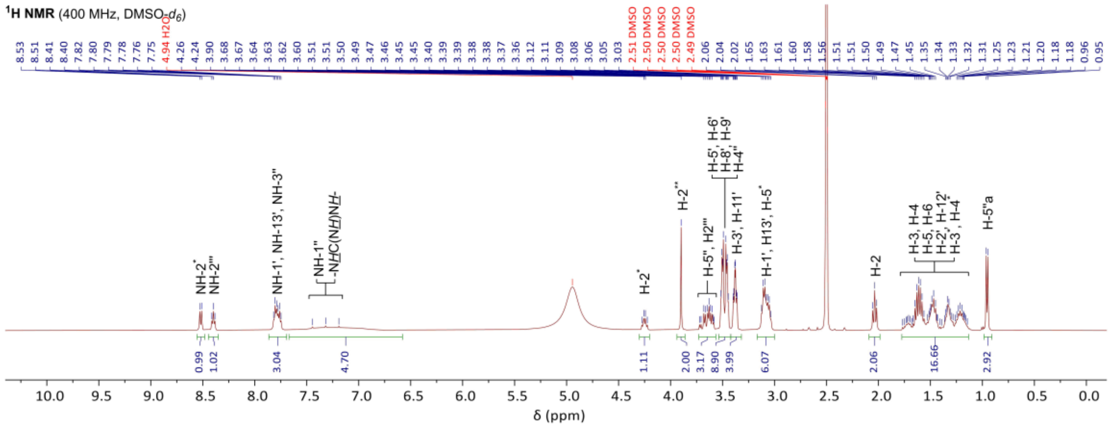

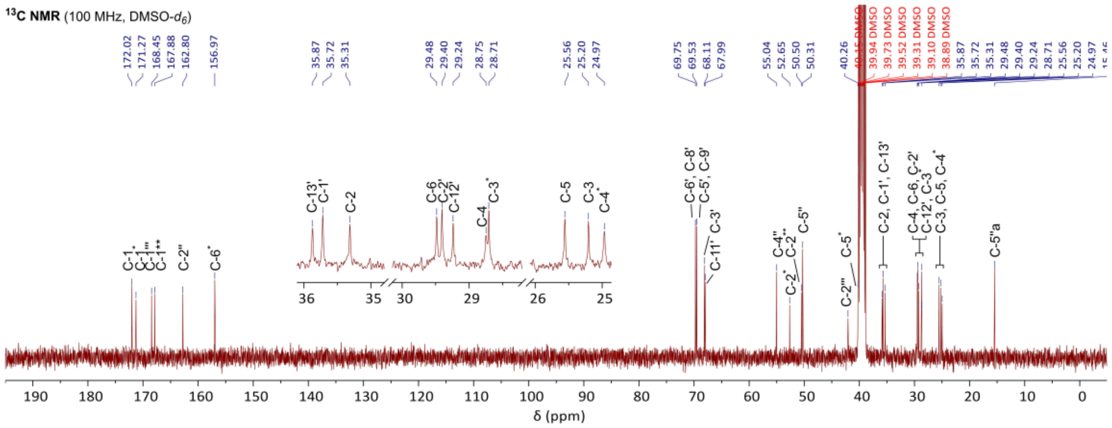

Supplementary Fig. 19

NMR characterisation of *N*-((*S*)-1-amino-6-(2-azidoacetamido)-1-imino-7,10-dioxo-15,18,21-trioxa-2,8,11-triazatetracosan-24-yl)-6-((4*R*,5*S*)-5-methyl-2-oxoimidazolidin-4-yl)hexanamide N_3-(Arg)PEG$_3$-DB.

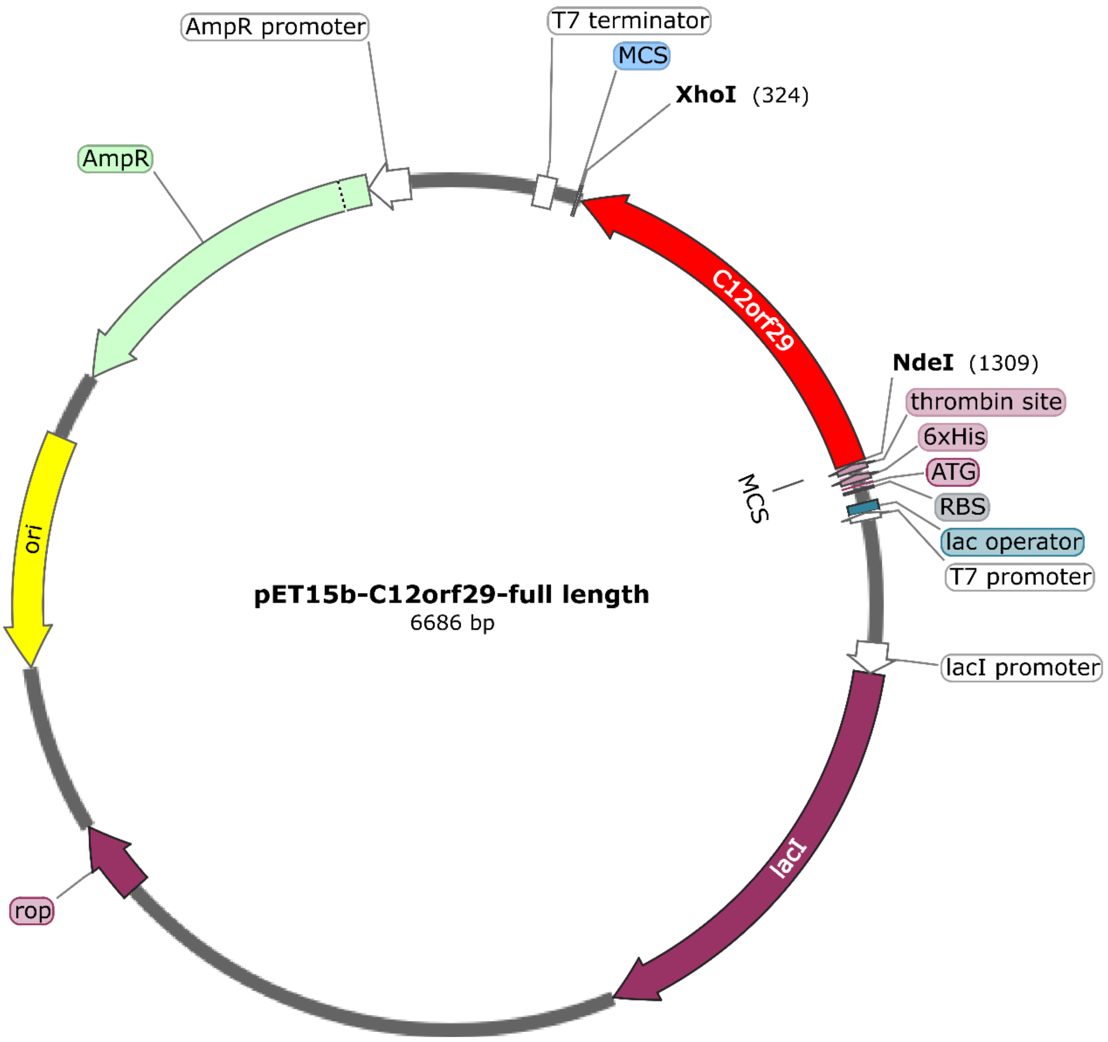

Supplementary Fig. 20

Plasmid map of pET15b-C12orf29^{WT}.

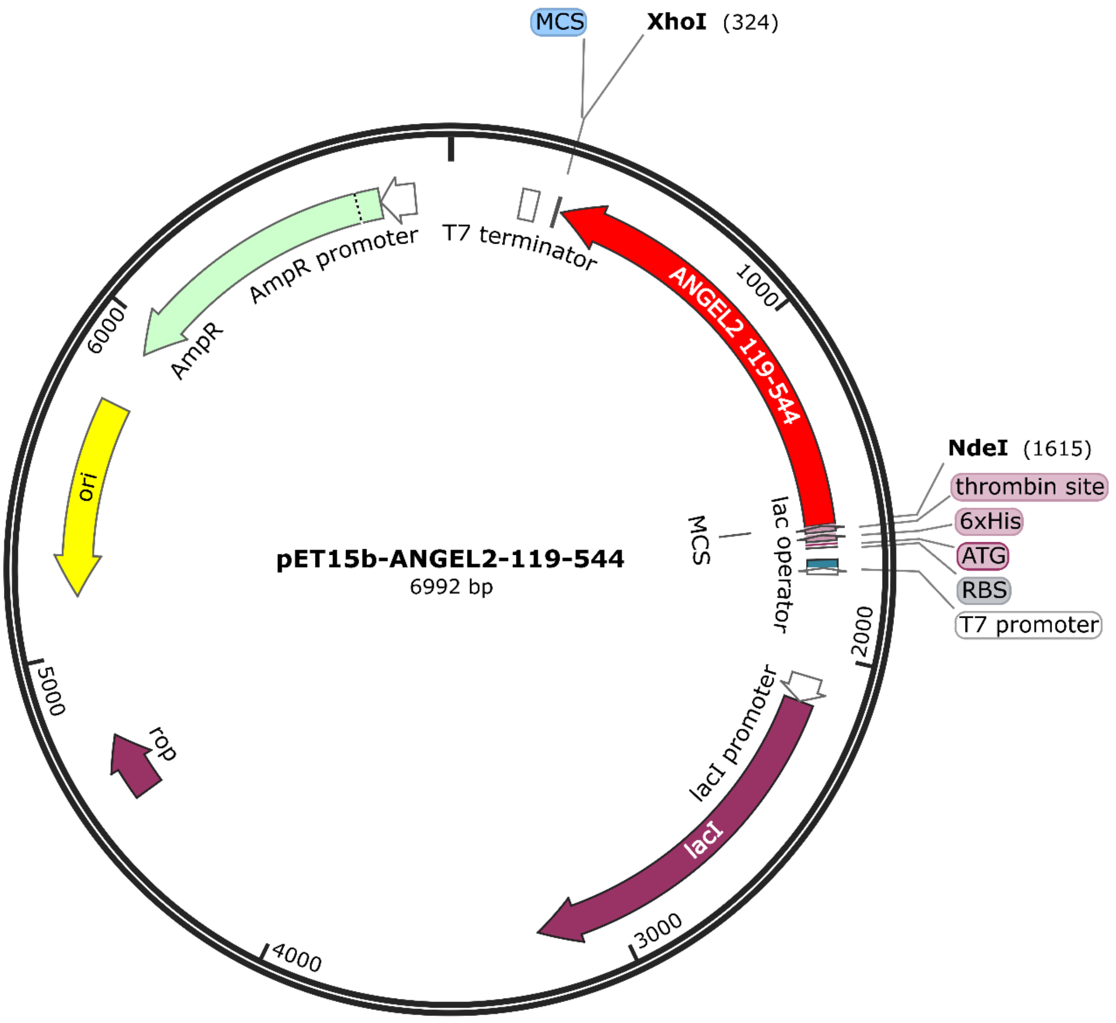

Supplementary Fig. 21

Plasmid map of pET15b-ANGEL2-ΔN.

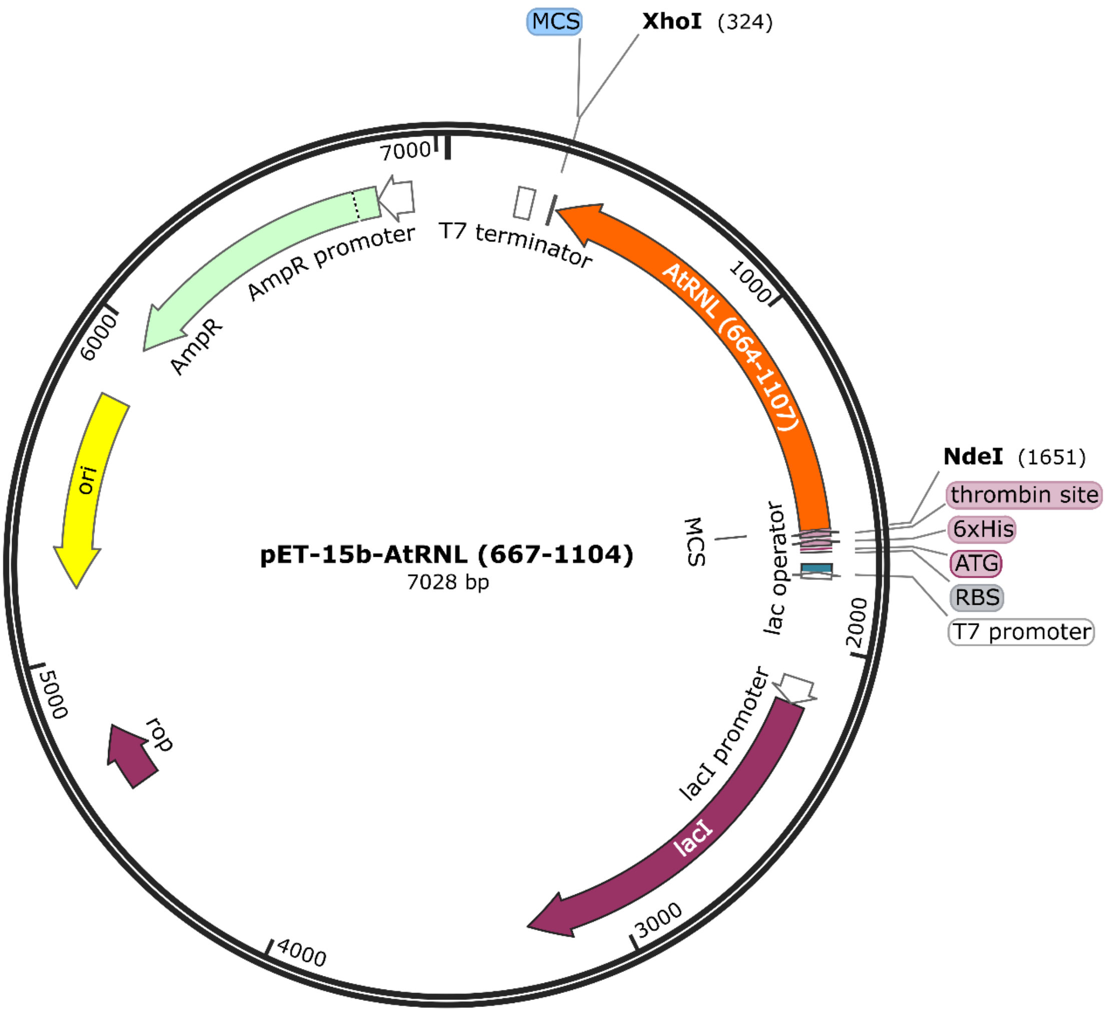

Supplementary Fig. 22
Plasmid map of pET15b-AtRNL-CPD.

Supplementary Tables

Oligonucleotide	Sequence (5' to 3')
RNA 1	GGC ACU CAG ACU CAG AG
RNA 2	CUC UGA GUA A
RNA 3	AGC ACU CAG ACU CAG ACU AGU AA
RNA 4	UUA CUA GUC UGA GUA A
RNA 5	AGC ACU CAG ACU AGU GAG AAC UAG UAA
RNA 6	CGC ACU CAG ACU AGU GAG AAC UAG UAA
RNA 7	GGC ACU CAG ACU AGU GAG AAC UAG UAA
RNA 8	UGC ACU CAG ACU AGU GAG AAC UAG UAA
RNA 9	AGC ACU CAG ACU AGU GAG AAC UAG UAC
RNA 10	CGC ACU CAG ACU AGU GAG AAC UAG UAC
RNA 11	GGC ACU CAG ACU AGU GAG AAC UAG UAC
RNA 12	UGC ACU CAG ACU AGU GAG AAC UAG UAC
RNA 13	AGC ACU CAG ACU AGU GAG AAC UAG UAG
RNA 14	CGC ACU CAG ACU AGU GAG AAC UAG UAG
RNA 15	GGC ACU CAG ACU AGU GAG AAC UAG UAG
RNA 16	UGC ACU CAG ACU AGU GAG AAC UAG UAG
RNA 17	AGC ACU CAG ACU AGU GAG AAC UAG UAU
RNA 18	CGC ACU CAG ACU AGU GAG AAC UAG UAU
RNA 19	GGC ACU CAG ACU AGU GAG AAC UAG UAU
RNA 20	UGC ACU CAG ACU AGU GAG AAC UAG UAU
RNA 21	AGC CAG ACU AGU GAG AAC UAG UAA
RNA 22	AGC ACU AGU GAG AAC UAG UAA
RNA 23	AGC ACU CAG ACU AGU AAA GCA CUC AGA CUA GUA A
DNA 1	GGC ACT CAG ACT CAG AG
DNA 2	CTC TGA GTA A

Supplementary Table 1

Oligonucleotide sequences used in this study.

Sample	WT 0 µM	KO 0 µM	WT 10 µM	KO 10 µM	WT 20 µM	KO 20 µM	WT 40 µM	KO 40 µM	WT 60 µM	KO 60 µM	WT 100 µM	KO 100 µM
Sample conc. [pg/µL]	5,200	4,360	6,120	4,500	3,910	4,750	4,680	4,460	4,470	4,000	4,800	4,240
18S [pg/µL]	841	724	905	680	614	802	727	968	799	884	938	1,120
28S [pg/µL]	3,040	2,210	3,500	2,260	1,950	2,050	2,220	984	1,970	900	745	540
18S/total RNA	0.16	0.17	0.15	0.15	0.16	0.17	0.16	0.22	0.18	0.22	0.20	0.26
28S/total RNA	0.58	0.51	0.57	0.50	0.50	0.43	0.47	0.22	0.44	0.23	0.16	0.13
28S/18S	3.60	3.00	3.90	3.30	3.20	2.60	3.10	1.00	2.50	1.00	0.80	0.50

Supplementary Table 2

RNA integrity analysis of total RNA of WT and KO HEK293 cells after menadione treatment. Data from the experiments in **Fig. 6** were shown, indicating the total concentrations of RNA, the concentrations of 18S and 28S rRNA, the ratios of 18S and 28S rRNA to the total RNA and the 28S/18S ratio. The RNA concentrations, as well as the 28S/18S ratio, were directly calculated by the TapeStation system. The ratios of 18S and 28S to total RNA were calculated respectively from the shown concentrations. Source data are provided as a Source Data file.

Supplementary Notes

Determination of ATP Level in Commercially Obtained GTP

To determine the ligation activity of C12orf29 with GTP in comparison to ATP, commercially obtained GTP was used (Jena Bioscience, NU-1012S, Lot: 102.079). To investigate potential ATP contamination in this GTP sample, quantification via mass spectrometry was conducted.

For this, an Agilent 6546 Q-TOF was utilised and coupled to an Agilent Infinity 1260 II HPLC system. For an exemplary experiment see Supplementary Fig. 23 below. The samples were eluted over a Hypercarb 100 x 2.1 mm, 5 µm column with a gradient of Solvent A (10 mM NH_4OAc + 0.1% diethylamine pH = 10.0) and Solvent B (MeCN). The gradient is depicted in Supplementary Table 3.

Time	Solvent A	Solvent B
0 min	95%	5%
2 min	95%	5%
15 min	0%	100%
20 min	0%	100%
25 min	95%	5%
30 min	95%	5%

Supplementary Table 3
Gradient for the quantification of ATP in commercially obtained GTP. Solvent A: 10 mM NH_4OAc + 0.1% diethylamine. Solvent B: MeCN.

Calibration curves at the start and at the end of the measurements were obtained by measuring ATP samples (Jena Bioscience, NU-1010S, Lot: 102.142) in different dilutions (50, 100, 250, and 500 nM). The area of the extracted ion chromatogram of ATP (505.9879 m/z ± 10 ppm) was linearly fitted. In-between, three different 50 µM GTP samples were measured in duplicates (10 µL injection) and the ATP content was determined by fitting the resulting signal of ATP to the calibration curve. Representative results were illustrated in Supplementary Fig. 5. By this, an ATP content of at most 0.2% of the total GTP concentration could be determined. This corresponds to a concentration of up to 0.4 µM ATP when using 200 µM GTP in experiments.

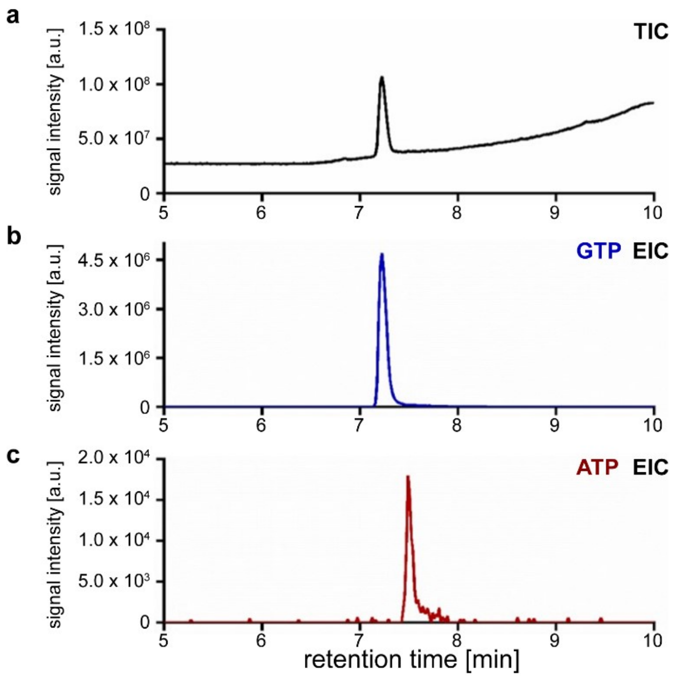

Supplementary Fig. 23

Determination of ATP in a commercial GTP sample by LC-MS. a, Total ion chromatogram (TIC) of an exemplary LC-MS quantification experiment for the quantification of ATP in a sample of 50 µM GTP. **b**, Extracted ion chromatogram (EIC) of GTP (521.9828 m/z ± 10 ppm). **c**, EIC of ATP (505.9879 m/z ± 10 ppm). Please note the about 100-fold difference in signal intensity in **b** (4.5×10^6) and **c** (2.0×10^4). a.u., arbitrary unit.

Supplementary Methods

Synthesis and Analysis of Chemical Probes

Chemicals and Reagents

Unless otherwise noted, all chemicals were purchased from commercial suppliers (abcr, Acros Organics, Fluka, Carl Roth, Sigma-Aldrich, TCI, and VWR) and used without further purification. Dry solvents were used in all reactions unless otherwise noted. DIPEA was dried over CaH_2, distilled, and stored over molecular sieves (4 Å) under N_2. Et_3N was dried over KOH, distilled, and stored over molecular sieves (4 Å) under N_2. The $CDCl_3$ and DMSO-*d6* were stored over molecular sieves (4 Å) under N_2 if applied in the NMR measurement of P^{III} compounds. For the preparation of aqueous solutions and buffers, ultra-pure water generated with the Merck Millipore BioPak® system (MilliQ® H_2O) was used.

General Remarks

NMR spectra were recorded by Bruker Avance III 400 MHz, 500 MHz, and 600 MHz spectrometers at room temperature. Chemical shifts (in ppm) were calibrated using residual undertreated solvent in $CDCl_3$, DMSO-*d6*, and D_2O as references, respectively. Multiplicities are denoted as s = singlet, d = doublet, t = triplet, q = quartet, quint = quintet, m = multiplet, dd = doublet of doublets, dt = doublet of triplets, ddd = doublet of doublet of doublets, br = broad. NMR data were analysed with MestReNova (version 14.1.2-25024). Low resolution MS (LR-MS) was performed by amazon SL (ESI-ion trap) from Bruker Daltonics. High resolution MS (HR-MS) were performed by micrOTOF II (ESI-TOF) from Bruker Daltonics.

Synthesis of Modified Ap3A

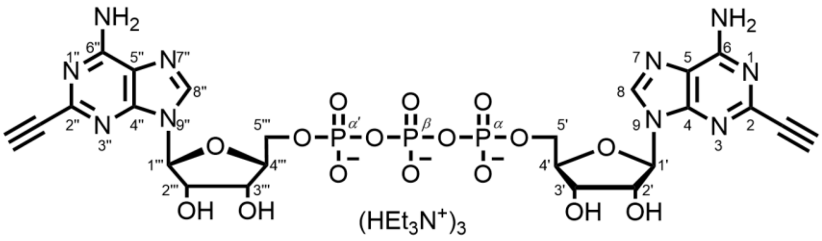

2,2''-Diethynyldiadenosine-*O*5',*O*5'''-triphosphate (triethylammonium salt form)

***C*2-eAp3A**

Compound **1**[6] as tetrabutylammonium salt (2.0 equiv, 0.050 mmol, 36.5 mg) was co-

evaporated with MeCN (3 × 3 mL) in an oven-dried 5 mL flask and dried under vacuum for 1 h. The compound was then dissolved in 0.5 mL DMF under N_2, followed by the addition of ETT (2.7 equiv, 67.5 µmol, 8.8 mg) and **2**[7] (1.0 equiv, 0.025 mmol, 10.7 mg). The resulting mixture was stirred at room temperature for 20 min, and $TBHP_{(dec)}$ (15.0 equiv, 0.375 mmol, 68.1 µL) was added in three portions. After stirring for 30 min, the reaction mixture was transferred to a falcon tube before the addition of Et_2O/*n*-hexane (5:1). The resulting suspension was centrifuged to give a pellet, which was washed with Et_2O/*n*-hexane (5:1) and centrifuged three times. After removing the supernatant, the pellet was dissolved in 0.5 mL DMF, and 50 µL piperidine was added to the solution. The reaction was stirred for 10 min at room temperature and was quenched by the addition of Et_2O/*n*-hexane (5:1). The suspension was centrifuged to give a pellet, which was washed with Et_2O/*n*-hexane (5:1) and centrifuged three times. The resulting pellet was purified via IEX-FPLC and RP-HPLC to give 9.7 mg ***C*2-eAp$_3$A** in 20% yield (UV absorption estimated). **^{1}H NMR** (400 MHz, D_2O) δ 8.38 (s, 2H, H-8, H-8''), 5.98 (d, *J* = 4.4 Hz, 2H, H-1', H-1'''), 4.63 (t, *J* = 4.6 Hz, 2H, H-2', H-2'''), 4.54 (t, *J* = 5.0 Hz, 2H, H-3', H-3'''), 4.41-4.34 (m, 6H, H-4', H-4''', H_a-5', H_a-5''', H_b-5', H_b-5'''), 3.59 (s, 2H, *C*2-C≡CH, *C*2''-C≡CH). **^{13}C NMR** (100 MHz, D_2O) δ 154.8 (C-6, C-6''), 148.3 (C-4, C-4''), 144.9 (C-2, C-2''), 140.2 (C-8, C-8''), 117.9 (C-5, C-5''), 87.4 (C-1', C-1'''), 83.2 (d, *J* = 9.0 Hz, C-4', C-4'''), 80.8 (*C*2-C≡CH, *C*2''-C≡CH), 76.3 (*C*2-C≡CH, *C*2''-C≡CH), 75.1 (C-2', C-2'''), 69.8 (C-3', C-3'''), 64.8 (d, *J* = 5.0 Hz, C-5', C-5'''). **^{31}P NMR** (162 MHz, D_2O) δ -11.52 (d, *J* = 19.4 Hz, 2P, P-α, P-α'), -23.04 (t, *J* = 19.4 Hz, 1P, P-β). **HR-MS** *m*/*z* calcd for $C_{24}H_{26}N_{10}O_{16}P_3^-$ $(M - H)^-$ 803.0747, found 803.0742, deviation 0.6 ppm.

Synthesis of N$_3$-(Arg)PEG$_3$-DB

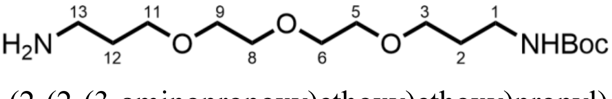

tert-Butyl (3-(2-(2-(3-aminopropoxy)ethoxy)ethoxy)propyl)carbamate **3**[8]

An oven-dried 250 mL two-neck flask was evacuated and flushed with N_2 trice, followed by the addition of 4,7,10-trioxa-1,13-tridecanediamine (5.2 equiv, 52.0 mmol, 11.38 mL) and 50 mL dioxane. A solution of di-*tert*-butyl dicarbonate (1.0 equiv, 10.0 mmol, 2.18 g) in 20 mL dioxane was added dropwise within 3 h at room temperature. The reaction mixture was stirred for additional 5 h and then concentrated. The residue was dissolved in 50 mL H_2O and extracted with DCM (4 × 50 mL). The organic phases were collected and washed with brine (4 × 30 mL). The extraction and the subsequent washing procedure were repeated four times. The organic phases were combined, dried over $MgSO_4$, concentrated and purified via silica chromatography (DCM:MeOH = 1:1) to give 2.94 g **3** as yellow oil in 92% yield. **^{1}H NMR** (400 MHz, $CDCl_3$) δ 5.11 (br s,

1H, NH-1), 3.63-3.60 (m, 4H, H-6, H-8), 3.58-3.56 (m, 4H, H-5, H-9), 3.54-3.50 (m, 4H, H-3, H-11), 3.22-3.17 (m, 2H, H-1), 2.81 (t, J = 6.6 Hz, 2H, H-13), 2.16 (br s, 2H, NH_2-13), 1.76-1.70 (m, 4H, H-2, H-12), 1.41 (s, 9H, -$C(CH_3)_3$). **^{13}C NMR** (100 MHz, $CDCl_3$) δ 156.2 (-<u>C</u>OO-), 79.0 (-<u>C</u>(CH_3)$_3$), 70.68, 70.65 (C-6, C-8), 70.31 (C-5), 70.26 (C-9), 69.64, 69.61 (C-3, C-11), 39.7 (C-13), 38.6 (C-1), 32.8 (C-12), 29.8 (C-2), 28.6 (-C(<u>C</u>H_3)$_3$). **LR-MS** *m*/*z* calcd for $C_{15}H_{33}N_2O_5^+$ $(M + H)^+$ 321.24, found 321.21.

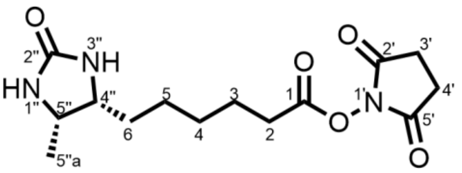

2,5-Dioxopyrrolidin-1-yl 6-((4*R*,5*S*)-5-methyl-2-oxoimidazolidin-4-yl)hexanoate **4**[9]

Desthiobiotin (1.0 equiv, 3.55 mmol, 761.2 mg), *N*-hydroxysuccinimide (1.0 equiv, 3.55 mmol, 408.7mg), and DCC (1.3 equiv, 4.62 mmol, 952.9 mg) were added to an oven-dried 50 mL flask, which was evacuated and flushed with N_2 trice, followed by the addition of 30 mL DMF. After stirring for 48 h at room temperature, the reaction mixture was filtered to remove formed *N*,*N*'-dicyclohexylurea. The solvent was evaporated, and 125 mL Et_2O was added. The suspension was filtered after stirring for 2 h at room temperature. The resulting white solid was recrystallized in 50 mL ^{i}PrOH to give 851.3 mg **4** as white powder in 77% yield. **^{1}H NMR** (400 MHz, DMSO-d_6) δ 6.29 (s, 1H, NH-3"), 6.11 (s, 1H, NH-1"), 3.61 (quint, J = 6.7 Hz, 1H, H-5"), 3.51-3.46 (m, 1H, H-4"), 2.81 (s, 4H, H-3', H-4'), 2.66 (t, J = 7.4 Hz, 2H, H-2), 1.62 (quint, J = 7.1 Hz, 2H, H-3), 1.42-1.18 (m, 6H, H-4, H-5, H-6), 0.96 (d, J = 6.0 Hz, 3H, H-5"a). **^{13}C NMR** (100 MHz, DMSO-d_6) δ 170.2 (C-2', C-5'), 169.0 (C-1), 162.7 (C-2"), 54.9 (C-4"), 50.2 (C-5"), 30.1 (C-2), 29.4 (C-6), 28.0 (C-4), 25.4 (C-3', C-4'), 25.3 (C-5), 24.2 (C-3), 15.5 (C-5"a). **HR-MS** *m*/*z* calcd for $C_{14}H_{22}N_3O_5^+$ $(M + H)^+$ 312.1559, found 312.1553, deviation 1.9 ppm.

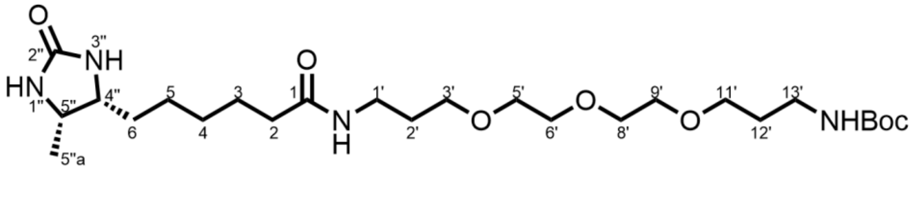

tert-Butyl (20-((4*R*,5*S*)-5-methyl-2-oxoimidazolidin-4-yl)-15-oxo-4,7,10-trioxa-14-azaicosyl)carbamate **5**[9]

Compound **4** (1.0 equiv, 3.19 mmol, 991.2 mg) and DMAP (0.1 equiv, 0.32 mmol, 39.0 mg) were added to an oven-dried 50 mL flask, which was evacuated and flushed with N_2 trice, followed by the addition of a solution of compound **3** (2.0 equiv, 6.38 mmol,

2.04 g) in 10 mL DMF and Et_3N (1.0 equiv, 3.19 mmol, 443.4 μL). After stirring overnight at room temperature, DMF was removed, and the residue was dissolved in 50 mL DCM. The solution was successively washed with 0.5 M HCl (2 × 25 mL), 1 M K_2CO_3 (2 × 25 mL), and brine (2 × 25 mL). The resulting organic phase was dried over $MgSO_4$, concentrated, and purified via silica chromatography (DCM:MeOH = 20:1) to give 1.58 g **5** as pale yellow oil in 96% yield. **^{1}H NMR** (400 MHz, DMSO-d_6) δ 7.70 (t, J = 5.6 Hz, 1H, NH-1'), 6.73 (t, J = 5.8 Hz, 1H, NH-13'), 6.28 (s, 1H, NH-3"), 6.10 (s, 1H, NH-1"), 3.60 (quint, J = 6.7 Hz, 1H, H-5"), 3.52-3.44 (m, 9H, H-5', H-6', H-8', H-9', H-4"), 3.40-3.36 (m, 4H, H-3', H-11'), 3.06 (q, J = 6.5 Hz, 2H, H-1'), 2.95 (q, J = 6.5 Hz, 2H, H-13'), 2.03 (t, J = 7.4 Hz, 2H, H-2), 1.63-1.55 (m, 4H, H-2', H-12'), 1.52-1.44 (m, 2H, H-3), 1.37 (s, 9H, -C(CH_3)$_3$), 1.35-1.16 (m, 6H, H-4, H-5, H-6), 0.95 (d, J = 6.4 Hz, 3H, H-5"a). **^{13}C NMR** (100 MHz, DMSO-d_6) δ 171.9 (C-1), 162.8 (C-2"), 155.5 (-<u>C</u>OO-), 77.4 (-<u>C</u>(CH_3)$_3$), 69.7 (C-6', C-8'), 69.5 (C-5', C-9'), 68.09, 68.06 (C-3', C-11'), 55.0 (C-4"), 50.2 (C-5"), 37.2 (C-13'), 35.7 (C-1'), 35.3 (C-2), 29.7 (C-12'), 29.5 (C-6), 29.4 (C-2'), 28.7 (C-4), 28.2 (-C(<u>C</u>H_3)$_3$), 25.5 (C-5), 25.2 (C-3), 15.5 (C-5"a). **HR-MS** *m/z* calcd for $C_{25}H_{49}N_4O_7^+$ $(M + H)^+$ 517.3601, found 517.3605, deviation 0.8 ppm.

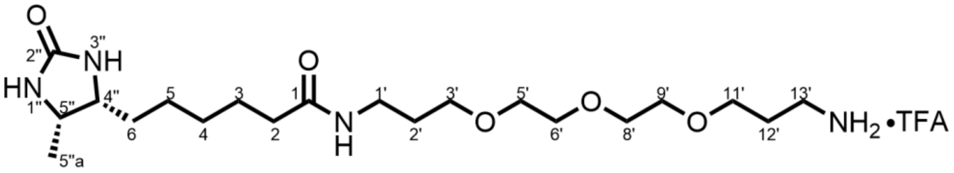

N-(3-(2-(2-(3-Aminopropoxy)ethoxy)ethoxy)propyl)-6-((4*R*,5*S*)-5-methyl-2-oxoimidazolidin-4-yl)hexanamide (trifluoroacetic acid salt form) **6**[9]

Compound **5** (1.0 equiv, 3.05 mmol, 1.57 g) was added to an oven-dried 50 mL flask, followed by the addition of 25 mL DCM. To the solution, 12.5 mL TFA was added slowly at 0 ºC. The reaction mixture was warmed up to room temperature and stirred for 2 h. Then DCM and TFA was removed under high vacuum overnight. The residue was purified via silica chromatography ($CHCl_3$:MeOH:$NH_{3(aq)}$ = 1:0.25:0.1) to give 1.40 g **6** as sticky yellow oil in 86% yield. **^{1}H NMR** (400 MHz, DMSO-d_6) δ 7.75 (t, J = 5.6 Hz, 1H, NH-1'), 6.29 (s, 1H, NH-3"), 6.12 (s, 1H, NH-1"), 4.67 (br s, 3H, NH_3^+-13'), 3.60 (quint, J = 6.7 Hz, 1H, H-5"), 3.53-3.44 (m, 11H, H-5', H-6', H-8', H-9', H-11', H-4"), 3.38 (t, J = 6.4 Hz, 2H, H-3'), 3.06 (q, J = 6.4 Hz, 2H, H-1'), 2.77 (t, J = 7.2 Hz, 2H, H-13'), 2.03 (t, J = 7.4 Hz, 2H, H-2), 1.71 (quint, J = 6.6 Hz, 2H, H-12'), 1.60 (quint, J = 6.7 Hz, 2H, H-2'), 1.47 (quint, J = 7.3 Hz, 2H, H-3), 1.36-1.16 (m, 6H, H-4, H-5, H-6), 0.95 (d, J = 6.4 Hz, 3H, H-5"a). **^{13}C NMR** (100 MHz, DMSO-d_6) δ 172.0 (C-1), 162.8 (C-2"), 158.0 (q, $^3J_{FC}$ = 30.7 Hz, -<u>C</u>OCF_3), 117.3 (q, $^2J_{FC}$ = 298.8 Hz, -<u>C</u>F_3), 69.74, 69.68 (C-6', C-8'), 69.52 (C-5'), 69.47 (C-9'), 68.1 (C-3'), 67.7 (C-11'), 55.0

(C-4''), 50.2 (C-5''), 37.4 (C-13'), 35.7 (C-1'), 35.3 (C-2), 29.5 (C-6), 29.4 (C-2'), 29.0 (C-12'), 28.7 (C-4), 25.6 (C-5), 25.2 (C-3), 15.5 (C-5''a). **^{19}F NMR** (376 MHz, DMSO-*d$_6$*) δ -73.6 (-CF_3). **HR-MS** *m/z* calcd for $C_{20}H_{41}N_4O_5^+$ $(M + H)^+$ 417.3077, found 417.3069, deviation 1.9 ppm.

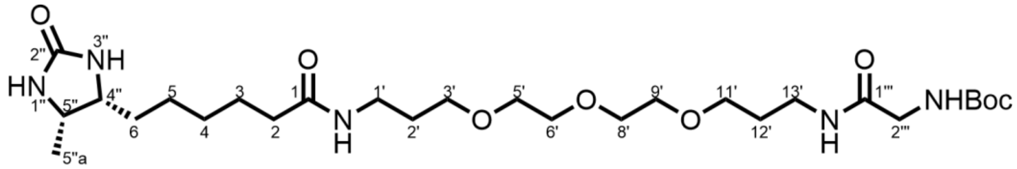

tert-Butyl (23-((4*R*,5*S*)-5-methyl-2-oxoimidazolidin-4-yl)-2,18-dioxo-7,10,13-trioxa-3,17-diazatricosyl)carbamate **7**

DMAP (0.1 equiv, 0.13 mmol, 15.9 mg) and Boc-Gly-NHS (1.5 equiv, 1.95 mmol, 530.6 mg) was added to an oven-dried 25 mL flask, which was evacuated and flushed with N_2 trice, followed by the addition of a solution of compound **6** (1.0 equiv, 1.30 mmol, 689.4 mg) in 10 mL DMF and Et_3N (2.5 equiv, 3.25 mmol, 451.7 μL). After stirring overnight at room temperature, DMF was removed, and the residue was dissolved in 50 mL DCM. The solution was washed with brine (1 × 25 mL), which was extracted with DCM (4 × 50 mL). The combined organic phases were dried over $MgSO_4$, concentrated, and purified via silica chromatography (DCM:MeOH = 20:1) to give 707.6 mg **7** as sticky colorless oil in 95% yield. **^{1}H NMR** (400 MHz, DMSO-*d$_6$*) δ 7.72-7.70 (m, 2H, NH-1', NH-13'), 6.87 (t, *J* = 6.2 Hz, 1H, NH-2'''), 6.28 (s, 1H, NH-3''), 6.10 (s, 1H, NH-1''), 3.60 (quint, *J* = 6.8 Hz, 1H, H-5''), 3.53-3.45 (m, 11H, H-5', H-6', H-8', H-9', H-4'', H-2'''), 3.40-3.36 (m, 4H, H-3', H-11'), 3.12-3.04 (m, 4H, H-1', H-13'), 2.03 (t, *J* = 7.4 Hz, 2H, H-2), 1.65-1.57 (m, 4H, H-2', H-12'), 1.47 (quint, *J* = 7.3 Hz, 2H, H-3), 1.38 (s, 9H, -$C(CH_3)_3$), 1.36-1.16 (m, 6H, H-4, H-5, H-6), 0.95 (d, *J* = 6.4 Hz, 3H, H-5''a). **^{13}C NMR** (100 MHz, DMSO-*d$_6$*) δ 171.9 (C-1), 169.1 (C-1'''), 162.8 (C-2''), 155.5 (-$\underline{C}$OO-), 78.0 (-$\underline{C}(CH_3)_3$), 69.74, 69.73 (C-6', C-8'), 69.5 (C-5', C-9'), 68.1 (C-3', C-11'), 55.0 (C-4''), 50.2 (C-5''), 43.3 (C-2'''), 35.9 (C-13'), 35.7 (C-1'), 35.3 (C-2), 29.5 (C-6), 29.4 (C-2'), 29.3 (C-12'), 28.7 (C-4), 28.2 (-$C(\underline{C}H_3)_3$), 25.6 (C-5), 25.2 (C-3), 15.5 (C-5''a). **HR-MS** *m/z* calcd for $C_{27}H_{52}N_5O_8^+$ $(M + H)^+$ 574.3810, found 574.3821, deviation 2.0 ppm.

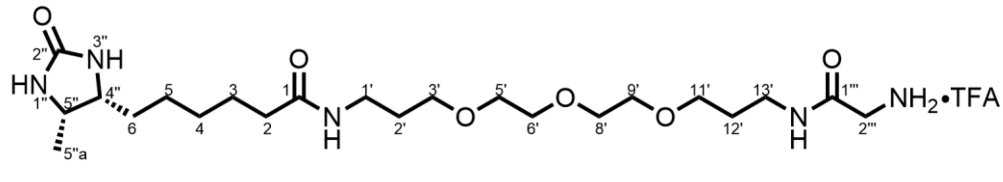

N-(1-Amino-2-oxo-7,10,13-trioxa-3-azahexadecan-16-yl)-6-((4*R*,5*S*)-5-methyl-2-oxoimidazolidin-4-yl)hexanamide (trifluoroacetic acid salt form) **8**

Compound **7** (1.0 equiv, 1.22 mmol, 697.0 mg) was added to an oven-dried 50 mL flask, followed by the addition of 10 mL DCM. To the solution, 5.0 mL TFA was added slowly at 0 °C. The reaction mixture was warmed up to room temperature and stirred for 2 h. Then DCM and TFA was removed under high vacuum overnight. The residue was purified via silica chromatography ($CHCl_3$:MeOH:$NH_{3(aq)}$ = 1:0.25:0.1) to give 568.3 mg of **8** sticky pale yellow oil in 80% yield. **^{1}H NMR** (400 MHz, DMSO-d_6) δ 7.86 (t, *J* = 5.8 Hz, 1H, NH-1'), 7.72 (t, *J* = 5.6 Hz, 1H, NH-13'), 6.28 (s, 1H, NH-3''), 6.10 (s, 1H, NH-1''), 3.60 (quint, *J* = 6.7 Hz, 1H, H-5''), 3.53-3.45 (m, 9H, H-5', H-6', H-8', H-9', H-4''), 3.42-3.36 (m, 4H, H-3', H-11'), 3.28 (br s, 3H, NH_3^+-2'''), 3.13 (q, *J* = 6.4 Hz, 2H, H-1'), 3.10 (s, 2H, H-2'''), 3.06 (q, *J* = 6.4 Hz, 2H, H-13'), 2.03 (t, *J* = 7.4 Hz, 2H, H-2), 1.67-1.57 (m, 4H, H-2', H-12'), 1.51-1.44 (m, 2H, H-3), 1.36-1.15 (m, 6H, H-4, H-5, H-6), 0.95 (d, *J* = 6.4 Hz, 3H, H-5''a). **^{13}C NMR** (100 MHz, DMSO-d_6) δ 171.93 (C-1), 171.85 (C-1'''), 162.8 (C-2''), 69.8 (C-6', C-8'), 69.53 (C-5', C-9'), 68.2 (C-3'), 68.1 (C-11'), 55.0 (C-4''), 50.2 (C-5''), 44.2 (C-2'''), 35.8 (C-13'), 35.7 (C-1'), 35.3 (C-2), 29.5 (C-6), 29.4 (C-2'), 29.3 (C-12'), 28.7 (C-4), 25.6 (C-5), 25.2 (C-3), 15.5 (C-5''a). ^{19}F NMR (376 MHz, DMSO-d_6) δ -73.5 (-CF_3). **HR-MS** *m/z* calcd for $C_{22}H_{44}N_5O_6^+$ $(M + H)^+$ 474.3286, found 474.3282, deviation 0.8 ppm.

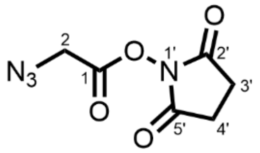

2,5-Dioxopyrrolidin-1-yl 2-azidoacetate **9**[10]

N-Hydroxysuccinimide (1.0 equiv, 4.50 mmol, 517.6 mg) was added to an oven-dried 25 mL flask, which was evacuated and flushed with N_2 trice, followed by the addition of 5.0 mL THF. After cooling down in ice bath, 2-azidoacetic acid (1.0 equiv, 4.50 mmol, 336.7 μL) was added and stirred for 10 min at 0 °C. DCC (1.0 equiv, 4.50 mmol, 928.5 mg) was suspended in 5.0 mL THF and added dropwise. The reaction mixture was stirred for 4 h at 0 °C, followed by filtration to remove formed *N,N'*-dicyclohexylurea. 50 mL Et_2O was added to the filtrate, which was stored at 4 °C overnight. The forming white solid was collected, rinsed with Et_2O (20 mL), and dried to give 669.0 mg of **9** as white crystal solid in 75% yield. **^{1}H NMR** (400 MHz, $CDCl_3$) δ 4.24 (s, 2H, H-2), 2.87 (s, 4H, H-3', H-4'). **^{13}C NMR** (100 MHz, $CDCl_3$) δ 168.5 (C-2', C-5'), 164.3 (C-1), 48.1 (C-2), 25.7 (C-3', C-4'). **HR-MS** *m/z* calcd for $C_6H_7N_4O_4^+$ $(M + H)^+$ 199.0467, found 199.0460, deviation 3.5 ppm.

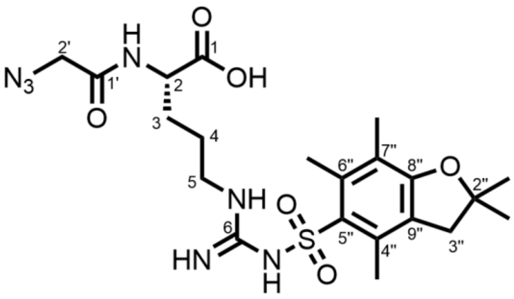

N^2-(2-Azidoacetyl)-N^ω-((2,2,4,6,7-pentamethyl-2,3-dihydrobenzofuran-5-yl)sulfonyl)-L-arginine **10**

In an oven-dried 50 mL flask, Fmoc-Arg(Pbf)-OH (1.0 equiv, 2.00 mmol, 1.298 g) was suspended in 30 mL DCM, followed by the addition of piperidine (5.0 equiv, 10.0 mmol, 987.8 μL). The reaction mixture was stirred for 16 h at room temperature and concentrated under reduced pressure. The resulting residue was added methyl *tert*-butyl ether (MTBE) to yield white precipitate, which was collected by filtration and washed with MTBE to give Fmoc deprotected intermediate Arg(Pbf)-OH. The white solid was dried under vacuum and used without further purification. DMAP (0.1 equiv, 0.20 mmol, 24.4 mg) and compound **52** (1.5 equiv, 3.00 mmol, 594.1 mg) was added to the flask containing Arg(Pbf)-OH, which was evacuated and flushed with N_2 trice, followed by the addition of 10 mL DMF and Et_3N (2.5 equiv, 5.00 mmol, 695.0 μL). After stirring overnight at room temperature, DMF was removed, and the residue was pre-purified via silica chromatography (DCM:MeOH = 20:1 ~ 3:1). Further RP-HPLC purification gave 472.2 mg **10** as pale yellow solid in 46% yield over two steps. **^{1}H NMR** (400 MHz, DMSO-*d$_6$*) δ 12.76 (br s, 1H, OH-1), 8.36 (d, *J* = 7.6 Hz, 1H, NH-2), 6.73-6.42 (m, 3H, -N*H*C(N*H*)N*H*-), 4.21-4.16 (m, 1H, H-2), 3.85 (s, 2H, H-2'), 3.04 (q, *J* = 6.5 Hz, 2H, H-5), 2.96 (s, 2H, H-3''), 2.48 (s, 3H, CH_3-6''), 2.42 (s, 3H, CH_3-4''), 2.01 (s, 3H, CH_3-7''), 1.76-1.67 (m, 1H, H_a-3), 1.60-1.51 (m, 1H, H_b-3), 1.45-1.41 (m, 2H, H-4), 1.41 (s, 6H, CH_3-2''). **^{13}C NMR** (100 MHz, DMSO-*d$_6$*) δ 173.0 (C-1), 167.4 (C-1'), 157.4 (C-8''), 156.1 (C-6), 137.2 (C-7''), 134.2 (C-5''), 131.4 (C-4''), 124.3 (C-9''), 116.3 (C-6''), 86.3 (C-2''), 51.8 (C-2), 50.4 (C-2'), 42.5 (C-3''), 39.6 (C-5, overlapped by DMSO-*d$_6$*), 28.4 (C-3), 28.3 (CH_3-2''), 25.6 (C-4), 18.9 (CH_3-4''), 17.6 (CH_3-6''), 12.3 (CH_3-7''). **HR-MS** *m*/*z* calcd for $C_{21}H_{30}N_7O_6S^-$ $(M - H)^-$ 508.1978, found 508.1985, deviation 1.4 ppm.

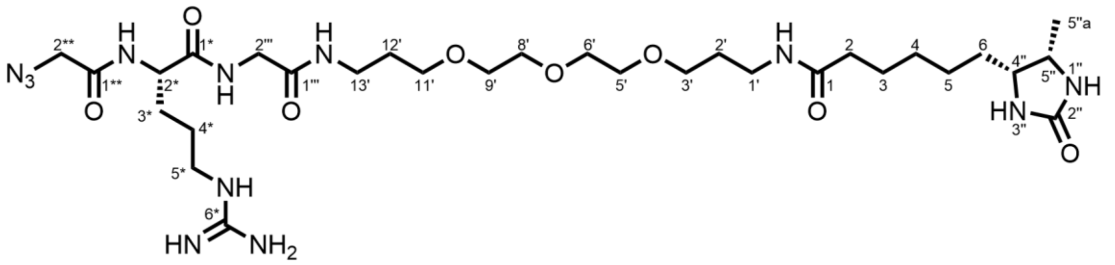

N-((*S*)-1-Amino-6-(2-azidoacetamido)-1-imino-7,10-dioxo-15,18,21-trioxa-2,8,11-triazatetracosan-24-yl)-6-((4*R*,5*S*)-5-methyl-2-oxoimidazolidin-4-yl)hexanamide

N_3-(Arg)PEG$_3$-DB

An oven-dried 10 mL flask was evacuated and flushed with N_2 trice, followed by the addition of compound **8** (1.0 equiv, 0.30 mmol, 1.10 mL 0.273 M solution in DMF) and Et_3N (1.0 equiv, 0.30 mmol, 41.7 μL). Meanwhile, compound **9** (2.0 equiv, 0.60 mmol, 305.5 mg) and HOBt·xH_2O (2.0 equiv, 0.60 mmol, 91.8 mg) was added to another oven-dried 10 mL flask, which was evacuated and flushed with N_2 trice, followed by the addition of 3.0 mL DMF. Then DCC (2.0 equiv, 0.60 mmol, 92.9 μL) was added and stirred for 15 min at room temperature, which was transferred to the flask containing compound **8** and Et_3N. After stirring for 3 h at room temperature, DMF was removed, and the residue was roughly purified via RP-HPLC. All fractions that contained Pbf-protected product were pooled, concentrated, and lyophilised. The crude product was then dissolved in 1 M HCl in HFIP and stirred for 8 h at room temperature to remove Pbf group. The reaction mixture was concentrated and purified via RP-HPLC with C18 Pyramid column using 0.1% TFA MilliQ® H_2O/0.1% TFA MeCN system. All fractions that contained Pbf-deprotected product were pooled, concentrated, and further purified via RP-HPLC with C18 column using MilliQ® H_2O /MeCN system to remove excess amount of TFA to give 126.6 mg of **N_3-(Arg)PEG$_3$-DB** as colorless oil in 59% yield over two steps. **^{1}H NMR** (400 MHz, DMSO-*d$_6$*) δ 8.52 (d, *J* = 7.2 Hz, 1H, NH-2*), 8.40 (t, *J* = 5.8 Hz, 1H, NH-2'''), 7.82-7.75 (m, 3H, NH-1', NH-13', NH-3''), 7.44-7.19 (m, 5H, NH-1'', -N*H*C(N*H*)N*H*$_2$), 4.25 (q, *J* = 6.8 Hz, 1H, H-2*), 3.90 (s, 2H, H-2**), 3.72-3.58 (m, 3H, H-5'', H-2'''), 3.51-3.45 (m, 9H, H-5', H-6', H-8', H-9', H-4''), 3.40-3.36 (m, 4H, H-3', H-11'), 3.12-3.03 (m, 6H, H-1', H-13', H-5*), 2.04 (t, *J* = 7.4 Hz, 2H, H-2), 1.77-1.15 (m, 16 H, H-3, H-4, H-5, H-6, H-2', H-12', H-3*, H-4*), 0.96 (d, *J* = 6.0 Hz, 3H, H-5''a). **^{13}C NMR** (100 MHz, DMSO-*d$_6$*) δ 172.0 (C-1), 171.3 (C-1*), 168.5 (C-1'''), 167.9 (C-1**), 162.8 (C-2''), 157.0 (C-6*), 69.8 (C-6', C-8'), 69.5 (C-5', C-9'), 68.1 (C-3'), 68.0 (C-11'), 55.0 (C-4''), 52.7 (C-2*), 50.5 (C-2**), 50.3 (C-5''), 42.1 (C-2'''), 40.3 (C-5*), 35.9 (C-13'), 35.7 (C-1'), 35.3 (C-2), 29.5 (C-6), 29.4 (C-2'), 29.2 (C-12'), 28.8 (C-4), 28.7 (C-3*), 25.6 (C-5), 25.2 (C-3), 25.0 (C-4*), 15.5 (C-5''a). **HR-MS** *m*/*z* calcd for $C_{30}H_{57}N_{12}O_8^+$ $(M + H)^+$ 713.4422, found 713.4422, deviation 0.0 ppm.

Plasmid Construction

The gene fragments of C12orf29^{WT}, AtRNL-CPD (667-1104), ANGEL2-ΔN (119-544), bearing 5'-NdeI and 3'-XhoI restriction cleavage sites were synthesised by Integrated DNA Technologies. Sequence information is listed in "Sequence Information" section in Supplementary Methods. The gene fragments were ligated into pJET1.2/blunt vector using CloneJET PCR Cloning Kit for amplification. The NdeI and XhoI restriction digested gene fragments and pET15b vector were isolated and ligated.

Site-directed Mutagenesis

50 ng plasmid DNA templates were mixed with 0.4 µM forward primers and 0.4 µM reverse primers in 1x Pfu reaction buffer with 0.4 mM dNTPs each and 2.5 units of Pfu Turbo DNA polymerase in a total volume of 50 µL. The reaction mixture was applied for PCR by the following program:

Step	Conditions	
Initial denaturation	95 °C, 2 min	
Denaturation	95 °C, 15 s	20 cycles
Primer annealing	55 °C, 30 s	
Extension	68 °C, 10 min	
Elongation	68 °C, 12 min	
Stopping	4 °C, pause	

Afterwards, 10 µL of PCR reaction mixture was incubated with 40 units of DpnI in 1x CutSmart® buffer for 30 min at 37 °C in a total volume of 50 µL. 10 µL of the resulting mixture was transformed into NEB Turbo chemically competent *E. coli* cells and plasmids were isolated for analysis.

Expression and Purification of Recombinant Proteins

Expression and Purification of Plant tRNA Ligase AtRNL

Plasmid constructs pET28a-At-TRL (Addgene: #32242) were transformed in *E. coli* BL21 (DE3) RIL competent cells, which were cultured in 50 mL LB medium containing 50 µg/mL kanamycin at 37 °C, 180 rpm overnight. In turn, a defined volume of cell suspension was transferred to 1 L LB medium containing 50 µg/mL kanamycin to reach OD_{600} = 0.1, followed by the incubation at 37 °C at 180 rpm until OD_{600} = 0.7. The mixture was cooled down on ice for 30 min and then incubated with 0.4 mM IPTG and 2% (v/v) ethanol at 17 °C for 20 h at 180 rpm. Cells were harvested by centrifugation at 4,400 rpm for 30 min at 4 °C. The pellet was resuspended in 30 mL cold lysis buffer (50 mM Tris-HCl pH 7.5, 250 mM NaCl, 10% (v/v) glycerol, 0.1% (v/v) Triton-X100, 20 mM imidazole, 1 µg/mL aprotinin, 1 µg/mL leupeptin, and 1 mg/mL Pefabloc® SC)

and lysed by sonication. The lysates were centrifuged at 40,000 x g for 30 min at 4 ºC and filtered through 0.45 µm syringe filter. The His_6-tagged AtRNL was purified using a 5 mL HisTrap™ FF crude column (Buffer A: 50 mM Tris-HCl pH 7.5, 250 mM NaCl, 10% (v/v) glycerol, and 20 mM imidazole; Buffer B: 50 mM Tris-HCl pH 7.5, 250 mM NaCl, 10% (v/v) glycerol, and 500 mM imidazole). Fractions containing His_6-tagged AtRNL were pooled and dialysed against a buffer containing 50 mM Tris-HCl pH 7.5, 250 mM NaCl, 5 U/mg thrombin, 10% glycerol, and 20 mM imidazole at 4 ºC overnight. The resulting solution was purified again using a 5 mL HisTrap™ FF crude column (Buffer A: 50 mM Tris-HCl pH 7.5, 250 mM NaCl, 10% (v/v) glycerol, and 20 mM imidazole; Buffer B: 50 mM Tris-HCl pH 7.5, 250 mM NaCl, 10% (v/v) glycerol, and 500 mM imidazole). The AtRNL were recovered in the flow-through, concentrated, and stored at -20 ºC in a storage buffer containing 25 mM Tris-HCl pH 8.0, 100 mM NaCl, and 50% (v/v) glycerol.

Expression and Purification of the Cyclic Phosphodiesterase Domain of Plant tRNA Ligase AtRNL (AtRNL-CPD)

Plasmid constructs pET15b-AtRNL-CPD were transformed in *E. coli* BL21 (DE3) competent cells, which were cultured in 50 mL LB medium containing 100 µg/mL carbenicillin at 37 ºC, 180 rpm overnight. In turn, a defined volume of cell suspension was transferred to 1 L LB medium containing 100 µg/mL carbenicillin to reach OD_{600} = 0.1, followed by the incubation at 37 ºC at 180 rpm until OD_{600} = 1.0. The mixture was cooled down on ice for 30 min and then incubated with 1.0 mM IPTG and 2% (v/v) ethanol at 20 ºC for 18 h at 180 rpm. Cells were harvested by centrifugation at 4,400 rpm for 30 min at 4 ºC. The pellet was resuspended in 30 mL cold lysis buffer (50 mM Tris-HCl pH 8.0, 200 mM NaCl, 10% (v/v) glycerol, 0.2% (v/v) Triton X-100, 20 mM imidazole, 1 µg/mL aprotinin, 1 µg/mL leupeptin, and 1 mg/mL Pefabloc® SC) and lysed by sonication. The lysates were centrifuged at 40,000 x g for 30 min at 4 ºC and filtered through 0.45 µm syringe filter. The His_6-tagged AtRNL-CPD was purified using a 5 mL HisTrap™ FF crude column (Buffer A: 50 mM Tris-HCl pH 8.0, 200 mM NaCl, 10% (v/v) glycerol, and 20 mM imidazole; Buffer B: 50 mM Tris-HCl pH 8.0, 200 mM NaCl, 10% (v/v) glycerol, and 500 mM imidazole). Fractions containing His_6-tagged AtRNL were pooled and dialysed against a buffer containing 50 mM Tris-HCl pH 8.0, 200 mM NaCl, 5 U/mg thrombin, 10% glycerol, and 20 mM imidazole at 4 ºC overnight. The resulting solution was purified again using a 5 mL HisTrap™ FF crude column (Buffer A: 50 mM Tris-HCl pH 8.0, 200 mM NaCl, 10% (v/v) glycerol, and 20 mM imidazole; Buffer B: 50 mM Tris-HCl pH 8.0, 200 mM NaCl, 10% (v/v) glycerol, and 500 mM imidazole). The AtRNL-CPD were recovered in the flow-through,

concentrated, and stored at -20 °C in a storage buffer containing 25 mM Tris-HCl pH 8.0, 100 mM NaCl, and 50% (v/v) glycerol.

Immunoblotting

Proteins were resolved by SDS-PAGE, transferred onto the MeOH-activated 0.2 μM PVDF membrane (Amersham™), and probed with anti-C12orf29 (Santa Cruz Biotechnology, sc-390730, 1:1,000), anti-p150 (Bioscience, 610474, 1:1,000) and anti-AMPylation antibodies[11] (1:1,000) kindly provided by Itzen Aymelt and colleagues, UKE, University of Hamburg. Anti-mouse antibody (Jackson ImmunoResearch, 115-035-062, 1:30,000) was used as secondary antibodies. Blots were developed using Pierce™ ECL Western Blotting Substrate (Thermo Fisher) or Pierece™ Fast Western Kit, SuperSignal™ West Pico (Thermo Fisher).

Preparation of Ligase Substrates

Preparation of Nicked RNA/DNA Duplexes

0.5 μM 5' ^{32}P-labelled RNA/DNA oligonucleotides were mixed with 1.0 μM DNA splint oligo and 2.5 μM of the second RNA/DNA oligo in 1x annealing buffer (10 mM Tris-HCl pH 6.8 and 200 mM NaCl) (Sequences are shown in Supplementary Table 1). The mixture was prepared freshly and applied for annealing by the following program:

Temperature	Time
65 °C	10 min
37 °C	15 min
22 °C	30 min
4 °C	pause

Preparation of RNA Oligo4 Bearing 2'-PO_4-3'-OH on the 3' Ends

0.5 μM RNA oligo4 bearing 2',3'-cPO_4 were incubated with 2.0 μM AtRNL-CPD in 50 mM Tris-HCl pH 7.5, 10 mM $MgCl_2$, and 2 mM DTT in a total volume of 4.0 mL. The reaction was split in a 96-well plate and incubated at 37 °C for 1.5 h. Each reaction was stopped by adding 5 μL pre-cold 50 mM EDTA pH 8.0 solution and heating at 95 °C for 2 min. All resulting mixtures were combined and purified with Oligo Clean & Concentrator Kit (Zymo Research).

Preparation of Broken RNA Hairpins

For preparing the complexes with 5'-PO_4 and 2'-OH-3'-OH overhangs, 0.5 μM 5' ^{32}P-labelled RNA were mixed with 2.5 μM RNA that donate 2'-OH-3'-OH ends in MilliQ®

H_2O. The mixture was prepared freshly and applied for the annealing program shown below.

For preparing the complexes with 5'-PO_4 and 2'-PO_4-3'-OH overhangs, 0.5 μM 5' ^{32}P-labelled RNA were mixed with 2.5 μM RNA that donate 2'-PO_4-3'-OH ends in MilliQ® H_2O. The mixture was prepared freshly and applied for the annealing program shown below.

For preparing the complexes with 5'-OH and 2',3'-cPO_4 overhangs, 0.5 μM 5' ^{32}P-labelled RNA oligo4 bearing 2',3'-cPO_4 ends were mixed with 2.5 μM RNA that donate 5'-OH ends in MilliQ® H_2O. The mixture was prepared freshly and applied for the annealing program shown below.

For preparing the complexes with 5'-PO_4 and 2',3'-cPO_4 overhangs, 0.5 μM 5' ^{32}P-labelled RNA oligo4 were mixed with 0.6 μM RNA oligo3 that donate 5'-PO_4 ends in MilliQ® H_2O. The mixture was prepared freshly and applied for the annealing program shown below.

The annealing step followed program:

Temperature	Time
95 °C	2 min
65 °C	30 s
55 °C	30 s
45 °C	30 s
25 °C	30 s
15 °C	30 s
10 °C	30 s
4 °C	pause

General Procedures of RNA Ligation Assays

General Procedure of RNA Ligation with T4 RNA Ligase 1

0.1 μM 5' ^{32}P-labelled oligonucleotide substrates were incubated with 5 units of T4 RNA ligase 1 (New England Biolabs, M0204S) and 1.0 mM ATP in 1x T4 RNA ligase reaction buffer with 20% PEG8000 at 25 °C for 2 h in a total volume of 10 μL. The reaction was quenched by adding 10 μL stopping solution and heating at 95 °C for 2 min. 1 μL of the resulting mixture was further diluted to give 0.005 μM 5' ^{32}P-labelled oligonucleotides. Samples were resolved by urea-PAGE and analysed by autoradiographic imaging.

General Procedure of RNA Ligation with T4 RNA Ligase 2

0.1 μM 5' ^{32}P-labelled oligonucleotide substrates were incubated with 5 units of T4 RNA ligase 2 in 1x T4 Rnl2 (New England Biolabs, M0239S) reaction buffer with 10 mM $MgCl_2$ at 37 °C 1 h in a total volume of 10 μL. The reaction was quenched by adding 10 μL stopping solution and heating at 95 °C for 2 min. 1 μL of the resulting mixture was further diluted to give 0.005 μM 5' ^{32}P-labelled oligonucleotides. Samples were resolved by urea-PAGE and analysed by autoradiographic imaging.

General Procedure of RNA Ligation with AtRNL

0.1 μM 5' ^{32}P-labelled oligonucleotide substrates were incubated with 5 μM AtRNL and 100 μM ATP in 1x RNA ligation buffer (50 mM Tris-HOAc pH 7.0, 5 mM $MgCl_2$, and 1 mM DTT) at 37 °C 1 h in a total volume of 10 μL. The reaction was quenched by adding 10 μL stopping solution and heating at 95 °C for 2 min. 1 μL of the resulting mixture was further diluted to give 0.005 μM 5' ^{32}P-labelled oligonucleotides. Samples were resolved by urea-PAGE and analysed by autoradiographic imaging.

General Procedure of RNA Ligation with RtcB

0.1 μM 5' ^{32}P-labelled oligonucleotide substrates were incubated with 1 μM RtcB (New England Biolabs, M0458S) and 100 μM GTP in 1x RtcB reaction buffer with 1 mM $MnCl_2$ at 37 °C 1 h in a total volume of 10 μL. The reaction was quenched by adding 10 μL stopping solution and heating at 95 °C for 2 min. 1 μL of the resulting mixture was further diluted to give 0.005 μM 5' ^{32}P-labelled oligonucleotides. Samples were resolved by urea-PAGE and analysed by autoradiographic imaging.

Nucleotide Screening in RNA Ligation Assay (Fig. 3b)

In the nucleotide screening study, 0.1 μM 5' ^{32}P-labelled RNA oligo1 and 0.5 μM RNA oligo2 were incubated with 1.0 μM C12orf29^{WT} and 200 μM of the indicated nucleotides in 1x RNA ligation buffer at 37 °C for 1 h in a total volume of 10 μL. The reaction was stopped by adding 150 μL stopping solution and heating at 95 °C for 2 min. 1 μL of samples were resolved by urea-PAGE and analysed by autoradiographic imaging.

Autoradiographic Analysis

Urea-PAGE Analysis and Phosphorimaging

Samples were quenched by adding stopping solution and denaturing at 95 °C for 2 min. 1 μL of denatured sample were loaded onto a pre-warmed 12% urea-PAGE sequencing gel. Electrophoresis was performed in 1x TBE buffer (90 mM Tris-HCl pH 8.0, 90 mM

boric acid, 2.0 mM EDTA) at constant power of 100 W. The gel was transferred onto Whatman® filter paper and dried at 80 °C for 2 h under vacuum. After cooling down to room temperature, the gel was exposed to a storage phosphor screen, of which the readout was performed by Typhoon™ FLA-9500 in phosphorimaging mode and further processed by Image Lab, Bio-Rad.

SDS-PAGE and Phosphorimaging

Samples were mixed with 6x loading buffer to a final concentration of 1x loading buffer and denatured at 95 °C for 5 min. Separation of proteins were realized by using 4% acrylamide stacking gel and 12.5% acrylamide resolving gel. As references an unstained or pre-stained protein ladder was used. Electrophoresis was performed in 1x SDS running buffer at constant voltage of 220 V for 40 min. The gel was stained by Coomassie staining solution at room temperature for 30 min after electrophoresis, followed by destaining, rinsing with MilliQ® H_2O, and imaging. In turn, the gel was soaked in the fixing solution (3% (v/v) glycerol, 20% (v/v) HOAc, 20% (v/v) MeOH) at room temperature for 30 min and rinsed in MilliQ® H_2O for 5 min before being transferred onto Whatman® filter paper and drying at 80 °C for 1 h under vacuum. After cooling down to room temperature, the gel was exposed to a storage phosphor screen, of which the readout was performed by Typhoon™ FLA-9500 in phosphorimaging mode and further processed by Image Lab, Bio-Rad.

Kinetic Analysis of C12orf29-catalysed RNA Ligation (Fig. 3c)

Steady state kinetics of C12orf29 were measured at different ATP or GTP concentrations. Nucleotide concentrations used were 30, 20, 10, 5.0, 2.0, 1.0, 0.5 and 0.1 µM for ATP and 200, 150, 100, 75, 50, 25, 10 and 2.0 µM for GTP. 0.1 µM ^{32}P-labelled RNA oligo1 and 0.5 µM RNA oligo2 were incubated with C12orf29^{WT} and the indicated concentrations of nucleotides in 1x RNA ligation buffer at 37 °C for 1 h in a total volume of 10 µL. The reaction was stopped by adding 150 µL stopping solution and heating at 95 °C for 2 min. 1 µL of samples were resolved by urea-PAGE and analysed by autoradiographic imaging. Enzymatic turnover was quantified by Image Lab. The parameters k_{cat}, K_M, and k_{cat}/K_M were determined from a non-linear regression fit/Michaelis-Menten curve of the initial rates at each substrate concentration using GraphPad Prism. The following equation was used. Plotted data represent the median value ± SD for three biological replicates.

$$v_0 = \frac{v_{max} * [S]}{K_M + [S]}$$

Mutational Effects on RNA Ligation

0.1 µM 5' ^{32}P-labelled RNA23 were applied in the standard RNA ligation assay with 1.0 µM C12orf29 variants. The readout was processed by Image Lab, Bio-Rad to give signal intensities, which were normalized to that of control experiment (without enzyme). The values represented arithmetic mean ± SD from three biological replicates in histogram.

Preparation of *C12ORF29* Knockout HEK293 Cell Lines

The *C12ORF29* knockout cell lines were generated in collaboration with trenzyme GmbH (Konstanz, Germany): gRNA oligonucleotides were designed to target and knock out *C12ORF29*. After successful nucleofection of the gRNA into the cells, single cell cloning by limiting dilution was performed. All resulting clones were picked and analysed resulting in clones that were homozygous for *C12ORF29* knockout. One of the knockout cell lines was chosen for further experiments.

Cell Viability Assay of HEK293 Cells Treated with Different Concentrations of Menadione

The CellTiter-Glo® Luminescent Cell Viability Assay (Promega) was used according to the manufacturer's instruction. 4.0 × 10^4 cells per well were seeded one day before the menadione treatment in 90 µL DMEM GlutaMAX™ medium with 10% FCS in a 96-well plate (Sarstedt). The plate was incubated at 37 °C, 5% CO_2, and 100% humidity for 24 h.

1,000x menadione stock solutions were prepared in ethanol and frozen in aliquots. On the day of the treatment, aliquots were thawed and diluted 1/100 in MilliQ® H_2O. The cells were treated with 10 µL of different concentrations of menadione in MilliQ® H_2O. The plate was incubated at 37 °C, 5% CO_2, 100% humidity. After 3 h, the cells were equilibrated to RT (15 min) and 100 µL of CellTiter-Glo® reagent was added per well and mixed thoroughly. The plate was incubated for 10 min on a shaker. 100 µL of the solution was transferred to a black 96-well plate and luminescence read out was performed with a plate reader (PerkinElmer Victor3™ Multilabel Counter 1420).

The luminescence values of the treated cells were compared to the value of the cells of the control treatment, which were treated with 0 µM menadione (equals 100% viability), to give the calculated cell viability.

Sequence Information

Unless otherwise noted, the thrombin-removable N-terminal regions are shaded in grey. The N-terminal His_6-tag regions are in italic. The thrombin recognition motifs are

underlined. The black arrows indicate the thrombin cleavage site. The starting codon and methionine in the protein coding region are in bold. The mutation sites are in red.

C12orf29^{WT}

Amino acid sequence:

MGSS*HHHHHH* SSGLVPR↓GSH

MKRLGSVQRK MPCVFVTEVK EEPSSKREHQ PFKVLATETV SHKALDADIY
SAIPTEKVDG TCCYVTTYKD QPYLWARLDR KPNKQAEKRF KNFLHSKENP
KEFFWNVEED FKPAPECWIP AKETEQINGN PVPDENGHIP GWVPVEKNNK
QYCWHSSVVN YEFEIALVLK HHPDDSGLLE ISAVPLSDLL EQTLELIGTN
INGNPYGLGS KKHPLHLLIP HGAFQIRNLP SLKHNDLVSW FEDCKEGKIE
GIVWHCSDGC LIKVHRHHLG LCWPIPDTYM NSRPVIINMN LNKCDSAFDI
KCLFNHFLKI DNQKFVRLKD IIFDV*

DNA sequence:

ATGGGCAGCAGC*CATCATCATCATCATCAC*AGCAGCGGCCTGGTGCCGC
GC↓GGCAGCCAT**ATG**AAGCGCCTTGGCAGCGTTCAACGCAAAATGCCT
TGTGTATTCGTCACAGAAGTCAAAGAAGAGCCTAGTAGCAAACGTGAG
CATCAGCCATTCAAGGTTTTGGCTACTGAGACGGTTTCCCACAAAGCG
TTGGACGCCGACATTTATTCGGCAATCCCTACGGAAAAAGTAGACGGA
ACCTGTTGCTACGTAACGACTTACAAAGACCAGCCTTATCTGTGGGCCC
GTTTAGACCGCAAACCCAATAAGCAGGCGGAGAAACGTTTTAAGAATT
TCCTTCATTCAAAAGAGAACCCTAAAGAGTTCTTTTGGAATGTCGAAG
AAGATTTTAAGCCCGCACCTGAATGTTGGATTCCCGCAAAAGAGACTG
AACAGATTAACGGGAATCCCGTACCGGACGAGAACGGCCATATCCCTG
GTTGGGTGCCCGTAGAGAAGAACAATAAACAATATTGCTGGCACAGCA
GCGTGGTGAACTACGAATTTGAGATTGCACTTGTTTTGAAGCACCACC
CAGATGATTCCGGCTTGTTGGAGATTAGCGCAGTACCGCTTAGTGACCT
TCTTGAACAGACCTTGGAGCTTATTGGTACAAACATCAACGGAAATCC
GTACGGTTTAGGCTCTAAGAAACATCCATTGCATCTTTTAATCCCGCAC
GGTGCCTTCCAAATCCGTAACTTACCTTCTCTTAAGCATAATGACTTAGT
ATCCTGGTTTGAAGATTGCAAGGAGGGGAAAATCGAAGGGATCGTCTG
GCACTGCAGCGACGGCTGCCTGATTAAAGTCCATCGCCACCATTTGGG
ACTGTGTTGGCCTATCCCAGACACGTATATGAATAGCCGCCCGGTTATTA
TTAACATGAATCTTAATAAGTGTGATAGCGCCTTCGATATCAAATGTCTT
TTTAACCATTTTTTAAAGATCGACAATCAAAAGTTTGTGCGCCTTAAAG
ATATCATTTTTGATGTATAATAA

C12orf29^{T55A}

Amino acid sequence:

MGSS*HHHHHH* SSGLVPR↓GSH

MKRLGSVQRK MPCVFVTEVK EEPSSKREHQ PFKVLATETV SHKALDADIY
SAIP**A**EKVDG TCCYVTTYKD QPYLWARLDR KPNKQAEKRF KNFLHSKENP
KEFFWNVEED FKPAPECWIP AKETEQINGN PVPDENGHIP GWVPVEKNNK
QYCWHSSVVN YEFEIALVLK HHPDDSGLLE ISAVPLSDLL EQTLELIGTN

INGNPYGLGS KKHPLHLLIP HGAFQIRNLP SLKHNDLVSW FEDCKEGKIE
GIVWHCSDGC LIKVHRHHLG LCWPIPDTYM NSRPVIINMN LNKCDSAFDI
KCLFNHFLKI DNQKFVRLKD IIFDV*

DNA sequence:
ATGGGCAGCAGC*CATCATCATCATCATCAC*AGCAGCGGCCTGGTGCCGC
GC↓GGCAGCCAT**ATG**AAGCGCCTTGGCAGCGTTCAACGCAAAATGCCT
TGTGTATTCGTCACAGAAGTCAAAGAAGAGCCTAGTAGCAAACGTGAG
CATCAGCCATTCAAGGTTTTGGCTACTGAGACGGTTTCCCACAAAGCG
TTGGACGCCGACATTTATTCGGCAATCCCT**GCG**GAAAAAGTAGACGGA
ACCTGTTGCTACGTAACGACTTACAAAGACCAGCCTTATCTGTGGGCCC
GTTTAGACCGCAAACCCAATAAGCAGGCGGAGAAACGTTTTAAGAATT
TCCTTCATTCAAAAGAGAACCCTAAAGAGTTCTTTTGGAATGTCGAAG
AAGATTTTAAGCCCGCACCTGAATGTTGGATTCCCGCAAAAGAGACTG
AACAGATTAACGGGAATCCCGTACCGGACGAGAACGGCCATATCCCTG
GTTGGGTGCCCGTAGAGAAGAACAATAAACAATATTGCTGGCACAGCA
GCGTGGTGAACTACGAATTTGAGATTGCACTTGTTTTGAAGCACCACC
CAGATGATTCCGGCTTGTTGGAGATTAGCGCAGTACCGCTTAGTGACCT
TCTTGAACAGACCTTGGAGCTTATTGGTACAAACATCAACGGAAATCC
GTACGGTTTAGGCTCTAAGAAACATCCATTGCATCTTTTAATCCCGCAC
GGTGCCTTCCAAATCCGTAACTTACCTTCTCTTAAGCATAATGACTTAGT
ATCCTGGTTTGAAGATTGCAAGGAGGGGAAAATCGAAGGGATCGTCTG
GCACTGCAGCGACGGCTGCCTGATTAAAGTCCATCGCCACCATTTGGG
ACTGTGTTGGCCTATCCCAGACACGTATATGAATAGCCGCCCGGTTATTA
TTAACATGAATCTTAATAAGTGTGATAGCGCCTTCGATATCAAATGTCTT
TTTAACCATTTTTTAAAGATCGACAATCAAAAGTTTGTGCGCCTTAAAG
ATATCATTTTTGATGTATAATAA

C12orf29^{K57A}

Amino acid sequence:
MGSS*HHHHHH* SSGLVPR↓GSH
MKRLGSVQRK MPCVFVTEVK EEPSSKREHQ PFKVLATETV SHKALDADIY
SAIPTE**A**VDG TCCYVTTYKD QPYLWARLDR KPNKQAEKRF KNFLHSKENP
KEFFWNVEED FKPAPECWIP AKETEQINGN PVPDENGHIP GWVPVEKNNK
QYCWHSSVVN YEFEIALVLK HHPDDSGLLE ISAVPLSDLL EQTLELIGTN
INGNPYGLGS KKHPLHLLIP HGAFQIRNLP SLKHNDLVSW FEDCKEGKIE
GIVWHCSDGC LIKVHRHHLG LCWPIPDTYM NSRPVIINMN LNKCDSAFDI
KCLFNHFLKI DNQKFVRLKD IIFDV*

DNA sequence:

ATGGGCAGCAGC*CATCATCATCATCATCAC*AGCAGCGGCCTGGTGCCGC
GC↓GGCAGCCAT**ATG**AAGCGCCTTGGCAGCGTTCAACGCAAAATGCCT
TGTGTATTCGTCACAGAAGTCAAAGAAGAGCCTAGTAGCAAACGTGAG
CATCAGCCATTCAAGGTTTTGGCTACTGAGACGGTTTCCCACAAAGCG
TTGGACGCCGACATTTATTCGGCAATCCCTACGGAA**GCG**GTAGACGGA
ACCTGTTGCTACGTAACGACTTACAAAGACCAGCCTTATCTGTGGGCCC
GTTTAGACCGCAAACCCAATAAGCAGGCGGAGAAACGTTTTAAGAATT
TCCTTCATTCAAAAGAGAACCCTAAAGAGTTCTTTTGGAATGTCGAAG
AAGATTTTAAGCCCGCACCTGAATGTTGGATTCCCGCAAAAGAGACTG
AACAGATTAACGGGAATCCCGTACCGGACGAGAACGGCCATATCCCTG
GTTGGGTGCCCGTAGAGAAGAACAATAAACAATATTGCTGGCACAGCA
GCGTGGTGAACTACGAATTTGAGATTGCACTTGTTTTGAAGCACCACC
CAGATGATTCCGGCTTGTTGGAGATTAGCGCAGTACCGCTTAGTGACCT
TCTTGAACAGACCTTGGAGCTTATTGGTACAAACATCAACGGAAATCC
GTACGGTTTAGGCTCTAAGAAACATCCATTGCATCTTTTAATCCCGCAC
GGTGCCTTCCAAATCCGTAACTTACCTTCTCTTAAGCATAATGACTTAGT
ATCCTGGTTTGAAGATTGCAAGGAGGGGAAAATCGAAGGGATCGTCTG
GCACTGCAGCGACGGCTGCCTGATTAAAGTCCATCGCCACCATTTGGG
ACTGTGTTGGCCTATCCCAGACACGTATATGAATAGCCGCCCGGTTATTA
TTAACATGAATCTTAATAAGTGTGATAGCGCCTTCGATATCAAATGTCTT
TTTAACCATTTTTTAAAGATCGACAATCAAAAGTTTGTGCGCCTTAAAG
ATATCATTTTTGATGTATAATAA

C12orf29^{D59A}

Amino acid sequence:

MGSS*HHHHHH* SSGLVPR↓GSH

MKRLGSVQRK MPCVFVTEVK EEPSSKREHQ PFKVLATETV SHKALDADIY
SAIPTEKV**A**G TCCYVTTYKD QPYLWARLDR KPNKQAEKRF KNFLHSKENP
KEFFWNVEED FKPAPECWIP AKETEQINGN PVPDENGHIP GWVPVEKNNK
QYCWHSSVVN YEFEIALVLK HHPDDSGLLE ISAVPLSDLL EQTLELIGTN
INGNPYGLGS KKHPLHLLIP HGAFQIRNLP SLKHNDLVSW FEDCKEGKIE
GIVWHCSDGC LIKVHRHHLG LCWPIPDTYM NSRPVIINMN LNKCDSAFDI
KCLFNHFLKI DNQKFVRLKD IIFDV*

DNA sequence:

ATGGGCAGCAGC*CATCATCATCATCATCAC*AGCAGCGGCCTGGTGCCGC
GC↓GGCAGCCAT**ATG**AAGCGCCTTGGCAGCGTTCAACGCAAAATGCCT
TGTGTATTCGTCACAGAAGTCAAAGAAGAGCCTAGTAGCAAACGTGAG
CATCAGCCATTCAAGGTTTTGGCTACTGAGACGGTTTCCCACAAAGCG
TTGGACGCCGACATTTATTCGGCAATCCCTACGGAAAAAGTA**GCC**GGA

ACCTGTTGCTACGTAACGACTTACAAAGACCAGCCTTATCTGTGGGCCC
GTTTAGACCGCAAACCCAATAAGCAGGCGGAGAAACGTTTTAAGAATT
TCCTTCATTCAAAAGAGAACCCTAAAGAGTTCTTTTGGAATGTCGAAG
AAGATTTTAAGCCCGCACCTGAATGTTGGATTCCCGCAAAAGAGACTG
AACAGATTAACGGGAATCCCGTACCGGACGAGAACGGCCATATCCCTG
GTTGGGTGCCCGTAGAGAAGAACAATAAACAATATTGCTGGCACAGCA
GCGTGGTGAACTACGAATTTGAGATTGCACTTGTTTTGAAGCACCACC
CAGATGATTCCGGCTTGTTGGAGATTAGCGCAGTACCGCTTAGTGACCT
TCTTGAACAGACCTTGGAGCTTATTGGTACAAACATCAACGGAAATCC
GTACGGTTTAGGCTCTAAGAAACATCCATTGCATCTTTTAATCCCGCAC
GGTGCCTTCCAAATCCGTAACTTACCTTCTCTTAAGCATAATGACTTAGT
ATCCTGGTTTGAAGATTGCAAGGAGGGGAAAATCGAAGGGATCGTCTG
GCACTGCAGCGACGGCTGCCTGATTAAAGTCCATCGCCACCATTTGGG
ACTGTGTTGGCCTATCCCAGACACGTATATGAATAGCCGCCCGGTTATTA
TTAACATGAATCTTAATAAGTGTGATAGCGCCTTCGATATCAAATGTCTT
TTTAACCATTTTTTAAAGATCGACAATCAAAAGTTTGTGCGCCTTAAAG
ATATCATTTTTGATGTATAATAA

C12orf29^{D59N}

Amino acid sequence:

MGSS*HHHHHH* SSGLVPR↓GSH

MKRLGSVQRK MPCVFVTEVK EEPSSKREHQ PFKVLATETV SHKALDADIY
SAIPTEKV**N**G TCCYVTTYKD QPYLWARLDR KPNKQAEKRF KNFLHSKENP
KEFFWNVEED FKPAPECWIP AKETEQINGN PVPDENGHIP GWVPVEKNNK
QYCWHSSVVN YEFEIALVLK HHPDDSGLLE ISAVPLSDLL EQTLELIGTN
INGNPYGLGS KKHPLHLLIP HGAFQIRNLP SLKHNDLVSW FEDCKEGKIE
GIVWHCSDGC LIKVHRHHLG LCWPIPDTYM NSRPVIINMN LNKCDSAFDI
KCLFNHFLKI DNQKFVRLKD IIFDV*

DNA sequence:

ATGGGCAGCAGC*CATCATCATCATCATCAC*AGCAGCGGCCTGGTGCCGC
GC↓GGCAGCCAT**ATG**AAGCGCCTTGGCAGCGTTCAACGCAAAATGCCT
TGTGTATTCGTCACAGAAGTCAAAGAAGAGCCTAGTAGCAAACGTGAG
CATCAGCCATTCAAGGTTTTGGCTACTGAGACGGTTTCCCACAAAGCG
TTGGACGCCGACATTTATTCGGCAATCCCTACGGAAAAAGTA**AAC**GGA
ACCTGTTGCTACGTAACGACTTACAAAGACCAGCCTTATCTGTGGGCCC
GTTTAGACCGCAAACCCAATAAGCAGGCGGAGAAACGTTTTAAGAATT
TCCTTCATTCAAAAGAGAACCCTAAAGAGTTCTTTTGGAATGTCGAAG
AAGATTTTAAGCCCGCACCTGAATGTTGGATTCCCGCAAAAGAGACTG
AACAGATTAACGGGAATCCCGTACCGGACGAGAACGGCCATATCCCTG

GTTGGGTGCCCGTAGAGAAGAACAATAAACAATATTGCTGGCACAGCA
GCGTGGTGAACTACGAATTTGAGATTGCACTTGTTTTGAAGCACCACC
CAGATGATTCCGGCTTGTTGGAGATTAGCGCAGTACCGCTTAGTGACCT
TCTTGAACAGACCTTGGAGCTTATTGGTACAAACATCAACGGAAATCC
GTACGGTTTAGGCTCTAAGAAACATCCATTGCATCTTTTAATCCCGCAC
GGTGCCTTCCAAATCCGTAACTTACCTTCTCTTAAGCATAATGACTTAGT
ATCCTGGTTTGAAGATTGCAAGGAGGGGAAAATCGAAGGGATCGTCTG
GCACTGCAGCGACGGCTGCCTGATTAAAGTCCATCGCCACCATTTGGG
ACTGTGTTGGCCTATCCCAGACACGTATATGAATAGCCGCCCGGTTATTA
TTAACATGAATCTTAATAAGTGTGATAGCGCCTTCGATATCAAATGTCTT
TTTAACCATTTTTTAAAGATCGACAATCAAAAGTTTGTGCGCCTTAAAG
ATATCATTTTTGATGTATAATAA

C12orf29^{R77L}

Amino acid sequence:

MGSS*HHHHHH* SSGLVPR↓GSH

MKRLGSVQRK MPCVFVTEVK EEPSSKREHQ PFKVLATETV SHKALDADIY
SAIPTEKVDG TCCYVTTYKD QPYLWA**L**LDR KPNKQAEKRF KNFLHSKENP
KEFFWNVEED FKPAPECWIP AKETEQINGN PVPDENGHIP GWVPVEKNNK
QYCWHSSVVN YEFEIALVLK HHPDDSGLLE ISAVPLSDLL EQTLELIGTN
INGNPYGLGS KKHPLHLLIP HGAFQIRNLP SLKHNDLVSW FEDCKEGKIE
GIVWHCSDGC LIKVHRHHLG LCWPIPDTYM NSRPVIINMN LNKCDSAFDI
KCLFNHFLKI DNQKFVRLKD IIFDV*

DNA sequence:

ATGGGCAGCAGC*CATCATCATCATCATCAC*AGCAGCGGCCTGGTGCCGC
GC↓GGCAGCCAT**ATG**AAGCGCCTTGGCAGCGTTCAACGCAAAATGCCT
TGTGTATTCGTCACAGAAGTCAAAGAAGAGCCTAGTAGCAAACGTGAG
CATCAGCCATTCAAGGTTTTGGCTACTGAGACGGTTTCCCACAAAGCG
TTGGACGCCGACATTTATTCGGCAATCCCTACGGAAAAAGTAGACGGA
ACCTGTTGCTACGTAACGACTTACAAAGACCAGCCTTATCTGTGGGCC**C**
TTTTAGACCGCAAACCCAATAAGCAGGCGGAGAAACGTTTTAAGAATT
TCCTTCATTCAAAAGAGAACCCTAAAGAGTTCTTTTGGAATGTCGAAG
AAGATTTTAAGCCCGCACCTGAATGTTGGATTCCCGCAAAAGAGACTG
AACAGATTAACGGGAATCCCGTACCGGACGAGAACGGCCATATCCCTG
GTTGGGTGCCCGTAGAGAAGAACAATAAACAATATTGCTGGCACAGCA
GCGTGGTGAACTACGAATTTGAGATTGCACTTGTTTTGAAGCACCACC
CAGATGATTCCGGCTTGTTGGAGATTAGCGCAGTACCGCTTAGTGACCT
TCTTGAACAGACCTTGGAGCTTATTGGTACAAACATCAACGGAAATCC
GTACGGTTTAGGCTCTAAGAAACATCCATTGCATCTTTTAATCCCGCAC

GGTGCCTTCCAAATCCGTAACTTACCTTCTCTTAAGCATAATGACTTAGT
ATCCTGGTTTGAAGATTGCAAGGAGGGAAAATCGAAGGATCGTCTG
GCACTGCAGCGACGGCTGCCTGATTAAAGTCCATCGCCACCATTTGGG
ACTGTGTTGGCCTATCCCAGACACGTATATGAATAGCCGCCCGGTTATTA
TTAACATGAATCTTAATAAGTGTGATAGCGCCTTCGATATCAAATGTCTT
TTTAACCATTTTTAAAGATCGACAATCAAAAGTTTGTGCGCCTTAAAG
ATATCATTTTTGATGTATAATAA

C12orf29^{E123D}

Amino acid sequence:

MGSS*HHHHHH* SSGLVPR↓GSH

MKRLGSVQRK MPCVFVTEVK EEPSSKREHQ PFKVLATETV SHKALDADIY
SAIPTEKVDG TCCYVTTYKD QPYLWARLDR KPNKQAEKRF KNFLHSKENP
KEFFWNVEED FKPAPECWIP AK**D**TEQINGN PVPDENGHIP GWVPVEKNNK
QYCWHSSVVN YEFEIALVLK HHPDDSGLLE ISAVPLSDLL EQTLELIGTN
INGNPYGLGS KKHPLHLLIP HGAFQIRNLP SLKHNDLVSW FEDCKEGKIE
GIVWHCSDGC LIKVHRHHLG LCWPIPDTYM NSRPVIINMN LNKCDSAFDI
KCLFNHFLKI DNQKFVRLKD IIFDV*

DNA sequence:

ATGGGCAGCAGC*CATCATCATCATCATCAC*AGCAGCGGCCTGGTGCCGC
GC↓GGCAGCCAT**ATG**AAGCGCCTTGGCAGCGTTCAACGCAAAATGCCT
TGTGTATTCGTCACAGAAGTCAAAGAAGAGCCTAGTAGCAAACGTGAG
CATCAGCCATTCAAGGTTTTGGCTACTGAGACGGTTTCCCACAAAGCG
TTGGACGCCGACATTTATTCGGCAATCCCTACGGAAAAAGTAGACGGA
ACCTGTTGCTACGTAACGACTTACAAAGACCAGCCTTATCTGTGGGCCC
GTTTAGACCGCAAACCCAATAAGCAGGCGGAGAAACGTTTTAAGAATT
TCCTTCATTCAAAAGAGAACCCTAAAGAGTTCTTTTGGAATGTCGAAG
AAGATTTTAAGCCCGCACCTGAATGTTGGATTCCCGCAAAA**GAC**ACTG
AACAGATTAACGGGAATCCCGTACCGGACGAGAACGGCCATATCCCTG
GTTGGGTGCCCGTAGAGAAGAACAATAAACAATATTGCTGGCACAGCA
GCGTGGTGAACTACGAATTTGAGATTGCACTTGTTTTGAAGCACCACC
CAGATGATTCCGGCTTGTTGGAGATTAGCGCAGTACCGCTTAGTGACCT
TCTTGAACAGACCTTGGAGCTTATTGGTACAAACATCAACGGAAATCC
GTACGGTTTAGGCTCTAAGAAACATCCATTGCATCTTTTAATCCCGCAC
GGTGCCTTCCAAATCCGTAACTTACCTTCTCTTAAGCATAATGACTTAGT
ATCCTGGTTTGAAGATTGCAAGGAGGGAAAATCGAAGGATCGTCTG
GCACTGCAGCGACGGCTGCCTGATTAAAGTCCATCGCCACCATTTGGG
ACTGTGTTGGCCTATCCCAGACACGTATATGAATAGCCGCCCGGTTATTA
TTAACATGAATCTTAATAAGTGTGATAGCGCCTTCGATATCAAATGTCTT

TTTAACCATTTTTTAAAGATCGACAATCAAAAGTTTGTGCGCCTTAAAG
ATATCATTTTTGATGTATAATAA

C12orf29^{E123Q}

Amino acid sequence:

MGSS*HHHHHH* SSGLVPR↓GSH

MKRLGSVQRK MPCVFVTEVK EEPSSKREHQ PFKVLATETV SHKALDADIY
SAIPTEKVDG TCCYVTTYKD QPYLWARLDR KPNKQAEKRF KNFLHSKENP
KEFFWNVEED FKPAPECWIP AK**Q**TEQINGN PVPDENGHIP GWVPVEKNNK
QYCWHSSVVN YEFEIALVLK HHPDDSGLLE ISAVPLSDLL EQTLELIGTN
INGNPYGLGS KKHPLHLLIP HGAFQIRNLP SLKHNDLVSW FEDCKEGKIE
GIVWHCSDGC LIKVHRHHLG LCWPIPDTYM NSRPVIINMN LNKCDSAFDI
KCLFNHFLKI DNQKFVRLKD IIFDV*

DNA sequence:

ATGGGCAGCAGC*CATCATCATCATCATCAC*AGCAGCGGCCTGGTGCCGC
GC↓GGCAGCCAT**ATG**AAGCGCCTTGGCAGCGTTCAACGCAAAATGCCT
TGTGTATTCGTCACAGAAGTCAAAGAAGAGCCTAGTAGCAAACGTGAG
CATCAGCCATTCAAGGTTTTGGCTACTGAGACGGTTTCCCACAAAGCG
TTGGACGCCGACATTTATTCGGCAATCCCTACGGAAAAAGTAGACGGA
ACCTGTTGCTACGTAACGACTTACAAAGACCAGCCTTATCTGTGGGCCC
GTTTAGACCGCAAACCCAATAAGCAGGCGGAGAAACGTTTTAAGAATT
TCCTTCATTCAAAAGAGAACCCTAAAGAGTTCTTTTGGAATGTCGAAG
AAGATTTTAAGCCCGCACCTGAATGTTGGATTCCCGCAAAA**CAG**ACTG
AACAGATTAACGGGAATCCCGTACCGGACGAGAACGGCCATATCCCTG
GTTGGGTGCCCGTAGAGAAGAACAATAAACAATATTGCTGGCACAGCA
GCGTGGTGAACTACGAATTTGAGATTGCACTTGTTTTGAAGCACCACC
CAGATGATTCCGGCTTGTTGGAGATTAGCGCAGTACCGCTTAGTGACCT
TCTTGAACAGACCTTGGAGCTTATTGGTACAAACATCAACGGAAATCC
GTACGGTTTAGGCTCTAAGAAACATCCATTGCATCTTTTAATCCCGCAC
GGTGCCTTCCAAATCCGTAACTTACCTTCTCTTAAGCATAATGACTTAGT
ATCCTGGTTTGAAGATTGCAAGGAGGGGAAAATCGAAGGGATCGTCTG
GCACTGCAGCGACGGCTGCCTGATTAAAGTCCATCGCCACCATTTGGG
ACTGTGTTGGCCTATCCCAGACACGTATATGAATAGCCGCCCGGTTATTA
TTAACATGAATCTTAATAAGTGTGATAGCGCCTTCGATATCAAATGTCTT
TTTAACCATTTTTTAAAGATCGACAATCAAAAGTTTGTGCGCCTTAAAG
ATATCATTTTTGATGTATAATAA

C12orf29^{E250A}

Amino acid sequence:

MGSS*HHHHHH* SSGLVPR↓GSH

MKRLGSVQRK MPCVFVTEVK EEPSSKREHQ PFKVLATETV SHKALDADIY

SAIPTEKVDG TCCYVTTYKD QPYLWARLDR KPNKQAEKRF KNFLHSKENP

KEFFWNVEED FKPAPECWIP AKETEQINGN PVPDENGHIP GWVPVEKNNK

QYCWHSSVVN YEFEIALVLK HHPDDSGLLE ISAVPLSDLL EQTLELIGTN

INGNPYGLGS KKHPLHLLIP HGAFQIRNLP SLKHNDLVSW FEDCKEGKI**A**

GIVWHCSDGC LIKVHRHHLG LCWPIPDTYM NSRPVIINMN LNKCDSAFDI

KCLFNHFLKI DNQKFVRLKD IIFDV*

DNA sequence:

ATGGGCAGCAGC*CATCATCATCATCATCAC*AGCAGCGGCCTGGTGCCGC

GC↓GGCAGCCAT**ATG**AAGCGCCTTGGCAGCGTTCAACGCAAAATGCCT

TGTGTATTCGTCACAGAAGTCAAAGAAGAGCCTAGTAGCAAACGTGAG

CATCAGCCATTCAAGGTTTTGGCTACTGAGACGGTTTCCCACAAAGCG

TTGGACGCCGACATTTATTCGGCAATCCCTACGGAAAAAGTAGACGGA

ACCTGTTGCTACGTAACGACTTACAAAGACCAGCCTTATCTGTGGGCCC

GTTTAGACCGCAAACCCAATAAGCAGGCGGAGAAACGTTTTAAGAATT

TCCTTCATTCAAAAGAGAACCCTAAAGAGTTCTTTTGGAATGTCGAAG

AAGATTTTAAGCCCGCACCTGAATGTTGGATTCCCGCAAAAGAGACTG

AACAGATTAACGGGAATCCCGTACCGGACGAGAACGGCCATATCCCTG

GTTGGGTGCCCGTAGAGAAGAACAATAAACAATATTGCTGGCACAGCA

GCGTGGTGAACTACGAATTTGAGATTGCACTTGTTTTGAAGCACCACC

CAGATGATTCCGGCTTGTTGGAGATTAGCGCAGTACCGCTTAGTGACCT

TCTTGAACAGACCTTGGAGCTTATTGGTACAAACATCAACGGAAATCC

GTACGGTTTAGGCTCTAAGAAACATCCATTGCATCTTTTAATCCCGCAC

GGTGCCTTCCAAATCCGTAACTTACCTTCTCTTAAGCATAATGACTTAGT

ATCCTGGTTTGAAGATTGCAAGGAGGGGAAAATC**GCA**GGGATCGTCTG

GCACTGCAGCGACGGCTGCCTGATTAAAGTCCATCGCCACCATTTGGG

ACTGTGTTGGCCTATCCCAGACACGTATATGAATAGCCGCCCGGTTATTA

TTAACATGAATCTTAATAAGTGTGATAGCGCCTTCGATATCAAATGTCTT

TTTAACCATTTTTTAAAGATCGACAATCAAAAGTTTGTGCGCCTTAAAG

ATATCATTTTTGATGTATAATAA

C12orf29^{E250Q}

Amino acid sequence:

MGSS*HHHHHH* SSGLVPR↓GSH

MKRLGSVQRK MPCVFVTEVK EEPSSKREHQ PFKVLATETV SHKALDADIY

SAIPTEKVDG TCCYVTTYKD QPYLWARLDR KPNKQAEKRF KNFLHSKENP

KEFFWNVEED FKPAPECWIP AKETEQINGN PVPDENGHIP GWVPVEKNNK

QYCWHSSVVN YEFEIALVLK HHPDDSGLLE ISAVPLSDLL EQTLELIGTN

INGNPYGLGS KKHPLHLLIP HGAFQIRNLP SLKHNDLVSW FEDCKEGKI**Q**
GIVWHCSDGC LIKVHRHHLG LCWPIPDTYM NSRPVIINMN LNKCDSAFDI
KCLFNHFLKI DNQKFVRLKD IIFDV*

DNA sequence:
ATGGGCAGCAGC*CATCATCATCATCATCAC*AGCAGCGGCCTGGTGCCGC
GC↓GGCAGCCAT**ATG**AAGCGCCTTGGCAGCGTTCAACGCAAAATGCCT
TGTGTATTCGTCACAGAAGTCAAAGAAGAGCCTAGTAGCAAACGTGAG
CATCAGCCATTCAAGGTTTTGGCTACTGAGACGGTTTCCCACAAAGCG
TTGGACGCCGACATTTATTCGGCAATCCCTACGGAAAAAGTAGACGGA
ACCTGTTGCTACGTAACGACTTACAAAGACCAGCCTTATCTGTGGGCCC
GTTTAGACCGCAAACCCAATAAGCAGGCGGAGAAACGTTTTAAGAATT
TCCTTCATTCAAAAGAGAACCCTAAAGAGTTCTTTTGGAATGTCGAAG
AAGATTTAAGCCCGCACCTGAATGTTGGATTCCCGCAAAAGAGACTG
AACAGATTAACGGGAATCCCGTACCGGACGAGAACGGCCATATCCCTG
GTTGGGTGCCCGTAGAGAAGAACAATAAACAATATTGCTGGCACAGCA
GCGTGGTGAACTACGAATTTGAGATTGCACTTGTTTTGAAGCACCACC
CAGATGATTCCGGCTTGTTGGAGATTAGCGCAGTACCGCTTAGTGACCT
TCTTGAACAGACCTTGGAGCTTATTGGTACAAACATCAACGGAAATCC
GTACGGTTTAGGCTCTAAGAAACATCCATTGCATCTTTTAATCCCGCAC
GGTGCCTTCCAAATCCGTAACTTACCTTCTCTTAAGCATAATGACTTAGT
ATCCTGGTTTGAAGATTGCAAGGAGGGGAAAATC**CAA**GGGATCGTCTG
GCACTGCAGCGACGGCTGCCTGATTAAAGTCCATCGCCACCATTTGGG
ACTGTGTTGGCCTATCCCAGACACGTATATGAATAGCCGCCCGGTTATTA
TTAACATGAATCTTAATAAGTGTGATAGCGCCTTCGATATCAAATGTCTT
TTTAACCATTTTTTAAAGATCGACAATCAAAAGTTTGTGCGCCTTAAAG
ATATCATTTTTGATGTATAATAA

C12orf29^{K263N}

Amino acid sequence:
MGSS*HHHHHH* SSGLVPR↓GSH
MKRLGSVQRK MPCVFVTEVK EEPSSKREHQ PFKVLATETV SHKALDADIY
SAIPTEKVDG TCCYVTTYKD QPYLWARLDR KPNKQAEKRF KNFLHSKENP
KEFFWNVEED FKPAPECWIP AKETEQINGN PVPDENGHIP GWVPVEKNNK
QYCWHSSVVN YEFEIALVLK HHPDDSGLLE ISAVPLSDLL EQTLELIGTN
INGNPYGLGS KKHPLHLLIP HGAFQIRNLP SLKHNDLVSW FEDCKEGKIE
GIVWHCSDGC LI**N**VHRHHLG LCWPIPDTYM NSRPVIINMN LNKCDSAFDI
KCLFNHFLKI DNQKFVRLKD IIFDV*

DNA sequence:

ATGGGCAGCAGC*CATCATCATCATCATCAC*AGCAGCGGCCTGGTGCCGC
GC↓GGCAGCCAT**ATG**AAGCGCCTTGGCAGCGTTCAACGCAAAATGCCT
TGTGTATTCGTCACAGAAGTCAAAGAAGAGCCTAGTAGCAAACGTGAG
CATCAGCCATTCAAGGTTTTGGCTACTGAGACGGTTTCCCACAAAGCG
TTGGACGCCGACATTTATTCGGCAATCCCTACGGAAAAAGTAGACGGA
ACCTGTTGCTACGTAACGACTTACAAAGACCAGCCTTATCTGTGGGCCC
GTTTAGACCGCAAACCCAATAAGCAGGCGGAGAAACGTTTTAAGAATT
TCCTTCATTCAAAAGAGAACCCTAAAGAGTTCTTTTGGAATGTCGAAG
AAGATTTTAAGCCCGCACCTGAATGTTGGATTCCCGCAAAAGAGACTG
AACAGATTAACGGGAATCCCGTACCGGACGAGAACGGCCATATCCCTG
GTTGGGTGCCCGTAGAGAAGAACAATAAACAATATTGCTGGCACAGCA
GCGTGGTGAACTACGAATTTGAGATTGCACTTGTTTTGAAGCACCACC
CAGATGATTCCGGCTTGTTGGAGATTAGCGCAGTACCGCTTAGTGACCT
TCTTGAACAGACCTTGGAGCTTATTGGTACAAACATCAACGGAAATCC
GTACGGTTTAGGCTCTAAGAAACATCCATTGCATCTTTTAATCCCGCAC
GGTGCCTTCCAAATCCGTAACTTACCTTCTCTTAAGCATAATGACTTAGT
ATCCTGGTTTGAAGATTGCAAGGAGGGGAAAATCGAAGGGATCGTCTG
GCACTGCAGCGACGGCTGCCTGATT**AAT**GTCCATCGCCACCATTTGGG
ACTGTGTTGGCCTATCCCAGACACGTATATGAATAGCCGCCCGGTTATTA
TTAACATGAATCTTAATAAGTGTGATAGCGCCTTCGATATCAAATGTCTT
TTTAACCATTTTTTAAAGATCGACAATCAAAAGTTTGTGCGCCTTAAAG
ATATCATTTTTGATGTATAATAA

AtRNL-CPD

Amino acid sequence:

MGSS*HHHHHH* SSGLVPR↓GSH

MQGVTPATPE PAVKEAVQKD EGLIVFFPGI PGSAKSALCK ELLNAPGGFG
DDRPVHTLMG DLVKGKYWPK VADERRKKPQ SIMLADKNAP NEDVWRQIED
MCRRTRASAV PIVADSEGTD TNPYSLDALA VFMFRVLQRV NHPGKLDKES
SNAGYVLLMF YHLYEGKNRN EFESELIERF GSLIKMPLLK SDRTPLPDPV
KSVLEEGIDL FNLHSRRHGR LESTKGTYAA EWTKWEKQLR DTLVANSEYL
SSIQVPFESM VHQVREELKT IAKGDYKPPS SEKRKHGSIV FAAINLPATQ
VHSLLEKLAA ANPTMRSFLE GKKKSIQEKL ERSHVTLAHK RSHGVATVAS
YSQHLNREVP VELTELIYND KMAALTAHVG SVDGETVVSK NEWPHVTLWT
AEGVTAKEAN TLPQLYLEGK ASRLVIDPPV SISGPLEFF*

DNA sequence:

ATGGGCAGCAGC*CATCATCATCATCATCAC*AGCAGCGGCCTGGTGCCGC
GC↓GGCAGCCAT**ATG**CAGGGTGTTACTCCTGCTACGCCTGAACCTGCT
GTAAAAGAGGCTGTTCAGAAAGATGAAGGTCTGATTGTGTTCTTCCCG

GGTATTCCGGGCTCCGCTAAGAGCGCTCTGTGTAAAGAACTGCTGAAC
GCACCGGGCGGTTTTGGTGATGACCGTCCGGTTCACACCCTGATGGGC
GACCTGGTGAAAGGTAAATACTGGCCTAAAGTTGCGGACGAGCGCCGC
AAGAAACCTCAGAGCATTATGCTGGCTGATAAAAACGCTCCTAACGAA
GATGTTTGGCGCCAGATTGAAGACATGTGTCGTCGTACCCGCGCATCTG
CAGTGCCGATTGTCGCGGATTCCGAAGGCACTGATACCAACCCGTACT
CCCTGGATGCGCTGGCTGTTTTCATGTTCCGCGTGCTGCAGCGTGTTAA
CCACCCAGGTAAACTGGACAAAGAAAGCTCCAACGCAGGTTATGTCCT
GCTGATGTTCTATCACCTGTATGAAGGTAAAAACCGTAACGAGTTCGAG
TCTGAACTGATCGAACGTTTTGGTTCCCTGATCAAAATGCCTCTGCTGA
AATCTGATCGTACTCCTCTGCCGGACCCAGTTAAATCTGTTCTGGAAGA
AGGCATTGACCTGTTTAATCTGCATAGCCGTCGCCACGGTCGTCTGGAA
TCTACCAAGGGCACTTACGCCGCGGAATGGACTAAGTGGGAGAAGCA
GCTGCGTGACACCCTGGTTGCTAACAGCGAATACCTGTCCTCCATCCAG
GTACCTTTCGAGTCCATGGTGCATCAGGTTCGCGAAGAACTGAAAACC
ATCGCCAAAGGCGACTACAAACCGCCATCTTCCGAAAAACGCAAACAT
GGCAGCATTGTTTTTGCGGCTATTAACCTGCCTGCCACTCAGGTTCACT
CCCTGCTGGAAAAACTGGCAGCGGCGAACCCGACCATGCGTTCTTTCC
TGGAAGGTAAAAAGAAATCTATCCAGGAAAAACTGGAACGTAGCCAC
GTGACGCTGGCGCACAAACGTTCCCACGGTGTGGCCACTGTGGCTTCC
TACTCTCAGCACCTGAACCGCGAAGTGCCAGTAGAACTGACCGAACTG
ATCTACAACGATAAAATGGCCGCGCTGACTGCTCACGTTGGTTCCGTTG
ACGGTGAAACTGTCGTGAGCAAAAACGAATGGCCTCACGTCACCCTGT
GGACCGCGGAAGGTGTTACCGCGAAAGAAGCGAACACCCTGCCGCAG
CTGTACCTGGAAGGTAAGGCTTCTCGTCTGGTAATCGACCCACCAGTCT
CTATCTCCGGTCCGCTGGAATTCTTCTAATAA

ANGEL2-ΔN

Amino acid sequence:

MGSS*HHHHHH* SSGLVPR↓GSH
MEPSSKRRKH QGVIKRNWEY ICSHDKEKTK ILGDKNVDPK CEDSENKFDF
SVMSYNILSQ DLLEDNSHLY RHCRRPVLHW SFRFPNILKE IKHFDADVLC
LQEVQEDHYG AEIRPSLESL GYHCEYKMRT GRKPDGCAIC FKHSKFSLLS
VNPVEFFRPD ISLLDRDNVG LVLLLQPKIP YAACPAICVA NTHLLYNPRR
GDIKLTQLAM LLAEISSVAH QKDGSFCPIV MCGDFNSVPG SPLYSFIKEG
KLNYEGLPIG KVSGQEQSSR GQRILSIPIW PPNLGISQNC VYEVQQVPKV
EKTDSDLTQT QLKQTEVLVT AEKLSSNLQH HFSLSSVYSH YFPDTGIPEV
TTCHSRSAIT VDYIFYSAEK EDVAGHPGAE VALVGGLKLL ARLSLLTEQD
LWTVNGLPNE NNSSDHLPLL AKFRLEL*

DNA sequence:

ATGGGCAGCAGC*CATCATCATCATCATCAC*AGCAGCGGCCTGGTGCCGC
GC↓GGCAGCCAT**ATG**GAACCGAGCAGCAAACGTCGTAAACATCAGGGT
GTTATTAAACGCAACTGGGAATATATCTGCAGCCACGATAAAGAGAAA
ACCAAAATTCTGGGCGATAAAAACGTTGATCCGAAATGTGAAGATAGC
GAGAACAAATTTGATTTTAGCGTGATGAGCTACAACATCCTGAGCCAG
GATCTGCTGGAAGATAATAGCCATCTGTATCGTCATTGTCGTCGTCCGGT
TCTGCATTGGAGCTTTCGTTTTCCGAACATTCTGAAAGAAATCAAACAC
TTCGATGCCGATGTTCTGTGTCTGCAAGAAGTTCAAGAGGATCATTATG
GTGCAGAAATTCGTCCGAGCCTGGAAAGCCTGGGTTATCATTGTGAGT
ATAAAATGCGTACCGGTCGTAAACCGGATGGTTGTGCAATTTGTTTTAA
ACACAGCAAATTTAGCCTGCTGAGCGTTAATCCGGTTGAATTTTTCCGT
CCGGATATTAGTCTGCTGGATCGTGATAATGTTGGTCTGGTTCTGCTGCT
GCAGCCGAAAATTCCGTATGCAGCATGTCCGGCAATTTGTGTTGCAAAT
ACCCATCTGCTGTATAATCCGCGTCGTGGTGATATTAAACTGACCCAGC
TGGCAATGCTGCTGGCAGAAATTAGCAGCGTTGCACATCAGAAAGATG
GTAGCTTTTGTCCGATTGTTATGTGCGGTGATTTTAACAGCGTTCCGGG
TAGTCCGCTGTATAGCTTTATCAAAGAAGGCAAACTGAATTACGAAGGT
CTGCCGATTGGTAAAGTTAGCGGTCAAGAACAGAGCAGCCGTGGTCAG
CGTATTCTGAGCATTCCGATTTGGCCTCCGAATCTGGGTATTAGCCAGA
ATTGTGTTTATGAAGTTCAGCAGGTTCCGAAAGTTGAGAAAACCGATA
GCGATCTGACCCAGACACAGCTGAAACAGACCGAAGTTCTGGTTACCG
CAGAAAAACTGAGCAGCAATCTGCAGCATCATTTTAGCCTGAGCAGCG
TTTATAGCCATTATTTTCCGGATACCGGCATTCCGGAAGTTACCACCTGT
CATAGCCGTAGCGCAATTACCGTTGATTATATCTTTTACAGCGCAGAGA
AAGAAGATGTTGCAGGTCATCCGGGTGCCGAAGTTGCACTGGTTGGTG
GTCTGAAACTGCTGGCACGTCTGAGTCTGCTGACCGAACAGGACCTGT
GGACCGTTAATGGCCTGCCGAATGAAAATAACAGCAGCGATCATCTGC
CGCTGCTGGCCAAATTTCGTCTGGAACTGTAATAA

Supplementary References

1. Goujon, M. et al. A new bioinformatics analysis tools framework at EMBL-EBI. *Nucleic Acids Res*. **38**, W695–W699 (2010).
2. Waterhouse, A.M., Procter, J.B., Martin, D.M.A, Clamp, M. & Barton, G. J. Jalview version 2—a multiple sequence alignment editor and analysis workbench. *Bioinformatics* **25**, 1189–1191 (2009).
3. Larsson, J. & Gustafsson, P. A case study in fitting area-proportional Euler diagrams with ellipses using eulerr. *SetVR@ Diagrams* 84–91 (2018).
4. Pei, J., Kim, B.-H. & Grishin, N. V. PROMALS3D: a tool for multiple protein sequence and structure alignments. *Nucleic Acids Res*. **36**, 2295–2300 (2008).
5. Broncel, M. et al. Multifunctional reagents for quantitative proteome-wide analysis of protein modification in human cells and dynamic profiling of protein lipidation during vertebrate development. *Angew. Chem. Int. Ed.* **54**, 5948–5951 (2015).
6. Wang, Y., Rösner, D., Grzywa, M. & Marx, A. Chain terminating and clickable NAD^+ analogues for labeling target proteins of ADP-ribosyltransferases. *Angew. Chem. Int. Ed*. **53**, 8159-8162 (2014).
7. Hofer, A. et al. A modular synthesis of modified phosphoanhydrides. *Chem. Eur. J.* **21**, 10116–10122 (2015).
8. Chen, X. et al. Site-selective azide incorporation into endogenous RNase A via a “chemistry” approach. *Org. Biomol. Chem.* **11**, 353–361 (2013).
9. Yang, D., Gao, Q., Xue, J., Deng, Z. & Lee, O. Y. Polycyclic epoxides and compositions thereof with anti-cancer activities. WO2016/181312 (2016).
10. Chandrika, N. T., Green, K. D., Houghton, J. L. & Garneau-Tsodikova, S. Synthesis and biological activity of mono- and di-*N*-acylated aminoglycosides *ACS Med. Chem. Lett.* **6**, 1134–1139 (2015).
11. Höpfner, D. et al. Monoclonal anti-AMP antibodies are sensitive and valuable tools for detecting patterns of AMPylation. *iScience* **23**, 101800 (2020).
